# Factors influencing inappropriate use of antibiotics in outpatient and community settings in China: a mixed-methods systematic review

**DOI:** 10.1136/bmjgh-2020-003599

**Published:** 2020-11-12

**Authors:** Leesa Lin, Ruyu Sun, Tingting Yao, Xudong Zhou, Stephan Harbarth

**Affiliations:** 1 Department of Infectious Disease Epidemiology, London School of Hygiene & Tropical Medicine, London, UK; 2 Institute of Social Medicine, School of Medicine, Zhejiang University, Hangzhou, China; 3 Infection Control Programme, University of Geneva Hospitals and Faculty of Medicine, Geneva, Switzerland; 4 Infectious Diseases Division, University of Geneva Hospitals and Faculty of Medicine, Geneva, Switzerland

**Keywords:** public health, respiratory infections, systematic review, health policy, health systems

## Abstract

**Background:**

For decades, antibiotics have been excessively consumed around the world, contributing to increased antimicrobial resistance (AMR) and negatively impacting health outcomes and expenditures. Antibiotic use in China accounts for half of worldwide antibiotic consumption, which mainly takes place in outpatient and community settings, and often unnecessarily for self-limiting community-acquired infections. This study aimed to identify and assess factors of inappropriate use of antibiotics in the Chinese context to inform the development of interventions to mitigate inappropriate consumption in the absence of clinical indications.

**Methods:**

We conducted a mixed-methods systematic review and included empirical studies with original data conducted in mainland China, Hong Kong and Taiwan that investigated factors of antibiotic use in the community including outpatient care among patients, caregivers and prescribers. We searched MEDLINE, EMBASE, the Cochrane Library, PsycINFO, Google Scholar and one Chinese database CNKI (China Knowledge Resource Integrated Database), using a combination of the key terms ‘antibiotic’, ‘antimicrobial’, ‘use’, ‘consumption’, ‘behaviour’, ‘prescribe’ and related syntax for all peer-reviewed publications published before June 2020. Health Belief Model was employed for data synthesis.

**Findings:**

Fifty-four studies were included in the full-text review: 44 quantitative, 5 qualitative and 5 mixed-methods studies. Despite a high AMR awareness, public perception/misconception of antibiotic efficacy and easy access to antibiotics for self-limiting conditions drive inappropriate demand and use in the community including primary care setting. Providers’ prescribing behaviours are influenced by financial incentives, lack of diagnostic capacity and concerns over complications.

**Conclusions:**

Inappropriate outpatient and community antibiotic use is influenced by non-biomedical factors at the individual, community, health system and societal levels in mainland China, contributing to a high antibiotic use rate. This study calls for context-tailored One Health interventions, restrictive antibiotic drug policy and multifaceted antibiotic stewardship programmes that simultaneously address drivers of inappropriate use from both the supply-side and demand-side within and beyond clinical settings.

**PROSPERO registration number:**

CRD42019139591.

**Key questions**

**What is already known?**

- Human use of antibiotics in China accounts for a quarter of worldwide antibiotic consumption, which mainly takes place in outpatient and community settings.
- Many of the antibiotic uses were inappropriate and often unnecessarily for self-limiting community-acquired infections.

**What are the new findings?**

- Inappropriate use of antibiotics is prevalent across China with significant regional variation influenced by non-biomedical factors within and beyond clinical settings.
- Multifaceted, interactive personal and environmental factors shape antibiotic use for both the supply-side and demand-side of China’s healthcare system.

**What do the new findings imply?**

- Inappropriate antibiotic consumption is unlikely to decrease without multifaceted, context-tailored strategies targeting patients, prescribers and healthcare systems.
- Future strategies should incorporate an evidence-based, context-tailored design that simultaneously addresses drivers of antibiotic misuse from both the supply-side and demand-side within and beyond clinical settings.

## Introduction

For decades, antibiotics have been excessively consumed around the world, contributing to increased antimicrobial resistance (AMR) and negatively impacting health outcomes and expenditures.^1–3^ Reducing inappropriate antibiotic use is a pressing global health priority. Human use of antibiotics in China accounts for a quarter of worldwide antibiotic consumption,^4 5^ which mainly takes place in outpatient and community settings, often unnecessarily for self-limiting community-acquired infections—mostly viral and non-complicated, and untreatable by antibiotics.^5 6^A thorough examination of the prevalence of and factors influencing community antibiotic use in China is vital to inform the development of relevant policy and intervention strategies aiming to mitigate inappropriate or unnecessary antibiotic use, namely antibiotics consumed in the absence of clinical indications. This study aimed to conduct a mixed-methods systematic review that identifies and assesses factors influencing healthcare users’ and providers’ antibiotic use in the Chinese context.

## Methods

This mixed-methods review aimed to identify determinants for inappropriate antibiotic use in the community, including primary care and hospital outpatient settings, in Mainland China, Hong Kong and Taiwan. We systematically searched the following databases: MEDLINE, EMBASE, the Cochrane Library, and PsycINFO, Google Scholar and one Chinese database CNKI (China Knowledge Resource Integrated Database), using a combination of the key terms ‘antibiotic’, ‘antimicrobial’, ‘use’, ‘consumption’, ‘behaviour’, ‘prescribe’ and related syntax for all peer-reviewed publications published before June 2020.

**Figure 1:**
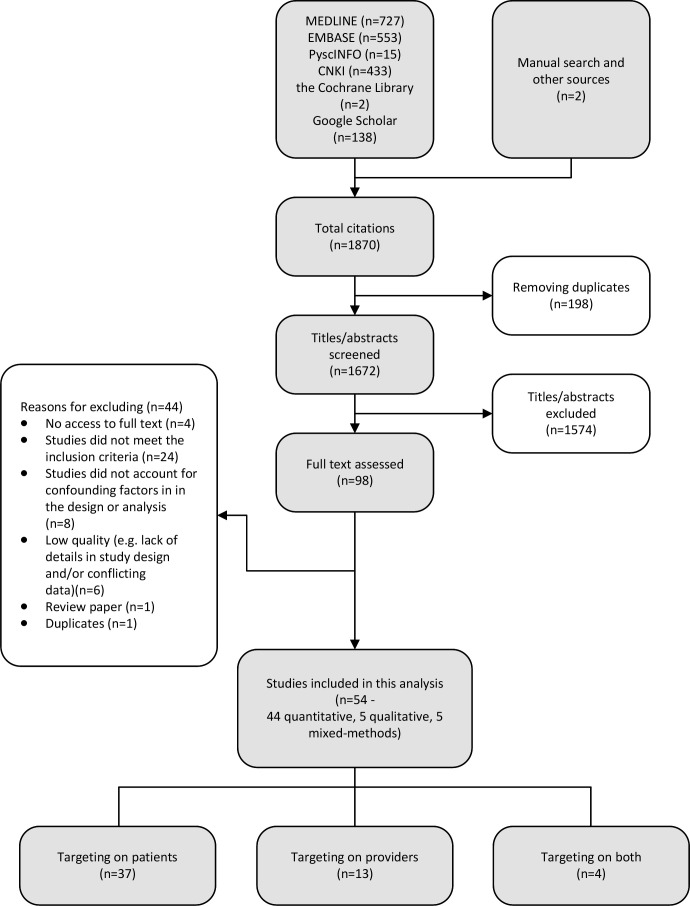
Flowchart of study identification and selection.

As a primary outcome of interest, ‘inappropriate antibiotic use’ included unnecessary antibiotic use for self-limiting viral infections (treatment or prophylaxis), self-medication with antibiotics by consumers and unnecessary antibiotic prescriptions for self-limiting viral infections by providers. Relevant behaviour outcomes, such as household storage of antibiotics, over-the-counter purchases and demands for antibiotic prescriptions, were also identified. No restrictions were applied to language, populations or antibiotic use for specific medical conditions. The search strategy for each database is presented in online supplemental file S1. Studies that focused only on (1) knowledge, attitudes and beliefs with regard to antibiotic use or (2) antibiotic prescriptions analysis were excluded. For the quantitative component, data from cross-sectional and longitudinal studies, where relevant confounders were accounted for by the study design or analysis, were included. Qualitative studies where methods of data collection and analysis were explicitly reported were eligible for inclusion. Experiments that generated empirical data were included whereas non-empirical studies or studies not reporting original data were excluded. A full list of inclusion/exclusion criteria is presented in online supplemental file S2. In addition, we conducted manual searches of the reference lists of included studies to identify additional relevant studies. All citations identified were imported to Endnote, and duplicates were deleted. Two reviewers (LL and TY or RS) independently screened titles and abstracts to select potentially relevant citations. Articles included in the full text review stage were retrieved and independently scrutinised. Any discrepancies in the process were resolved through discussion with a third reviewer until consensus was reached (see figure 1).

10.1136/bmjgh-2020-003599.supp1 Supplementary data

A standardised form based on Cochrane Review and behavioural theories including the Health Belief Model^7^ and Social Ecological Framework^8^ was developed specifically for this review prior to data extraction. Data were double extracted by two reviewers (TY and RS). Employing the Health Belief Model, we aimed to identify factors that could explain and predict individual uptake of antibiotics while adopting the Socio Ecological Framework, we incorporated the complex interplay between individual, relationship, community and societal factors in our synthesis and analysis of data.^9^ Disagreements were discussed with a third reviewer (LL) and resolved through consensus. The information extracted included characteristics of the study, methods, target population, sample size, antibiotic use behaviours and associated factors influencing behaviours. Numerical data (numbers or percentages) that reported prevalence and non-medical factors of antibiotic use were extracted from the quantitative component; themes relevant to factors influencing antibiotic use behaviours were extracted for the qualitative component.

### Quality assessment of included studies

Three reviewers (LL, TY, RS) independently assessed the risk of bias in all included studies using predetermined tools and reached consensus through discussion when discrepancies arose. The quantitative studies and quantitative components from mixed-methods studies that met inclusion criteria were assessed by adapted *BMJ* survey appraisal tools^10^; qualitative studies and the qualitative components from mixed-methods studies were appraised by the Critical Appraisals Skills Programme Appraisal Checklists^11^; experiments and mixed-methods studies were appraised by Mixed Methods Appraisal Tool.^12^ We followed the Preferred Reporting Items for Systematic Reviews and Meta-Analyses statement guidelines for reporting systematic reviews in structuring the review findings.

### Patient and public involvement statement

Patient and public were not involved in this systematic literature review.

## Results

We identified 54 studies: 37 focused on the consumers of healthcare, 13 on providers and 4 on both, involving a total of 104 619 participants. Table 1 summarised the characteristics of the included studies. We noted seven experiments employed the simulated client/patient method (SCM/SPM) to investigate factors influencing healthcare providers’ antibiotic use and only one study took the One Health approach investigating associations between antibiotic use for human and animals (ie, farm pigs). Almost all studies (n=53; 98.1%) employed cross-sectional designs and all included adult participants, with some (n=9; 16.7%) specifically involving the parents of children. More than half (n=30; 55.6%) of the included studies were published after 2016. There were 44 quantitative (including 6 experiments), 5 qualitative and 5 mixed-methods studies (including 1 experiment). Nine studies were conducted in Hong Kong, one in Taiwan and the rest (n=44) in mainland China, a majority covering both rural and urban settings. Little evidence about community antibiotic use was available from Taiwan. Identified non-biomedical factors of antibiotic use in the community were analysed and synthesised, presented in table 2.

**Table 1:** Summary of characteristics of included studies that investigated non-biomedical factors influencing outpatient and community antibiotic use in China

Characteristic	Number of studies	Studies
Total	**54**	13–36 38–63 73–76
Language		
Chinese	11	29 33 35 41 42 44 46 49 62 63 76
English	43	13–28 30–32 34 36 38–40 43 45 47 48 50–61 73–75
Year of study		
2001–2005	2	26 39
2006–2010	3	15 33 58
2011–2015	19	16 18 21 23 24 29–32 34 40–44 47 49 57 73
2016–later	30	13 14 17 19 20 22 25 27 28 35 36 38 45 46 48 50–56 59–63 74–76
Study design		
Quantitative study	44	13–15 17–26 28–30 33–36 38–55 62 63 73–76
Longitudinal	1	53
Cross-sectional	38	13–15 17–22 26 28–30 33–36 38–52 62 63 73–76
Experiment	6	23–25 53–55
Qualitative study	5	27 57–59 61
Mixed-methods	5	16 31 32 56 60
Experiment	1	56
Study region		
*Mainland China*		
East	14	23 24 34 35 40–42 46 48 49 52 60 62 63
Central	7	14 18 27 47 61 75 76
West	6	22 29 43 44 58 59
Across regions	17	13 17 19 20 25 28 36 38 45 50 51 53–57 74
*Hong Kong*	9	15 16 21 26 30–32 39 73
*Taiwan*	1	33
Urbanicity		
Urban	10	28 33 34 41 42 55 61 62 73 76
Rural	7	14 25 27 52 57 59 60
Both	33	13 15–24 26 29–32 35 36 38 39 43–47 49–51 53 54 56 58 63
Unknown	4	40 48 74 75
Participants		
*Healthcare consumers*		
General public (adults >18 years)	29	14–17 19–21 29–36 38 40 43 44 46–50 52 57 62 63 73
Parents or caregivers	8	13 18 28 41 42 45 51 76
*Healthcare providers*		
Healthcare professionals and/or community pharmacies	13	22–26 39 53–55 60 61 74 75
*Both sides of healthcare system*	4	27 56 58 59
Antibiotic misuse in the community		
Self-medication with antibiotics	34	13–21 27–36 40–46 48–52 58 62 63
Taking antibiotics as prophylaxis	12	13 17–20 36 38 43 50–52 57
Over-the-counter purchases/sales	22	15 16 18 21 28 30–33 41 44 48 50 52–59 76
Patient side reported	19	15 16 18 21 28 30–33 41 44 48 50 52 56–59 76
Experiment	4	53–56
Household storage of antibiotics	23	13 14 16–21 28 29 31 32 35 41–43 46 50–52 58 62 63
Demand for antibiotic prescriptions	23	13 16–21 27–36 38 47 57 62 63 76

**Table 2:** Non-biomedical factors influencing outpatient and community antibiotic use for common community-acquired infections

Non-biomedical factors	Application/examples	Inappropriate antibiotic use (including prevention use)	
		Antibiotic use behaviour outcomes	References
**Knowledge**			
General knowledge about antibiotics/AMR	Combined knowledge score Inadequate diagnostic knowledge of doctors Misconceptions (eg, antibiotic is an anti-inflammatory drug)	Asking/pressuring doctors for antibiotics Self-medication with antibiotics Storing antibiotics at home Taking antibiotics as prophylaxis Healthcare seeking behaviour The likelihood to be prescribed with antibiotics by doctors Combined behaviour score	14 17 25 36 46 63
Literacy	Being able to recognise antibiotics Knowing when/how to use antibiotics	No evidence available to date	–
Knowledge about the infection	The participant’s knowledge about the specific infection (eg, URTI symptoms will dissipate naturally)	No evidence available to date	–
AMR awareness	The participant’s awareness of AMR as a health threat on individual or on the society as a whole	No evidence available to date	–
**Attitudes**			
Attitudes towards antibiotic misuse behaviours	The participant’s accepting attitudes towards storing/self-medicating with antibiotics	Self-medication with antibiotics Storing antibiotics at home	28 52
Self-efficacy	The participant’s perception of his/her or others’ competence in engaging in caring for the infection or in antibiotic use	No evidence available to date	–
Medical background	The participants or their family members having some level of medical education	Asking/pressuring doctors for antibiotics Self-medication with antibiotics Over-the-counter purchases Storing antibiotics at home Taking antibiotics as prophylaxis The likelihood to be prescribed with antibiotics by doctors Combined behaviour score	13 19 20 28 34 40 41 43 44 47–51
Prior experience	Participants use of antibiotics on previous occasions	Over-the-counter purchase	76
**Perceptions**			
Perceived susceptibility	Self-rated health status	Self-medication with antibiotics Combined behaviour score	28 46 63
Perceived severity	The participant’s assessment/perception of the severity of the situation regarding the infection (eg, self-diagnosed symptoms experienced) The participant’s perception of potential harm of over-the-counter purchase	Over-the-counter purchase	32
Perceived benefits and disbenefits	The participant’s mistaken understanding of antibiotics (eg, considering antibiotics as Xiaoyanyao, anti-inflammatory drugs) (misconceptions)	Asking/pressuring doctors for antibiotics Self-medication with antibiotics Taking antibiotics as prophylaxis	36
Perceived barriers	The participant’s assessment/perception of barriers to engaging in antibiotic use (health insurance and knowledge of current policy)	Self-medication with antibiotics Healthcare seeking behaviour	14 28
Family dynamics	Family members who might influence the healthcare decisions of caregiver or the patients	Self-medication with antibiotics	28 45
Doctor–patient relationships	Having a regular doctor Following all the advice from physicians	Asking/pressuring doctors for antibiotics Self-medication with antibiotics	18 30
**Access to antibiotics**	Access to antibiotics, with or without prescription		
Access to non-prescription antibiotics	Over-the-counter purchase Antibiotics stored at home Leftover prescriptions	Self-medication with antibiotics Taking antibiotics as prophylaxis	17 18 28 31 41 42 46 50 51
Access to antibiotic prescriptions	Asking/pressuring doctors for antibiotics The education level, training, specialty or seniority of the doctors	The likelihood to be prescribed with antibiotics by doctors	22–24
**Cues to action**	External trigger mechanisms to prompt engagement in antibiotic use behaviour		
Symptoms	Presence of fever	No evidence available to date	–
Information sources and seeking for therapeutic purposes decisions	Expectation for antibiotic use knowledge	Combined behaviour score	63
**Socio-contextual factors**			
Age	The age of the participant or caregiver	Asking/pressuring doctors for antibiotics Self-medication with antibiotics Over-the-counter purchase Storing antibiotics at home Taking antibiotics as prophylaxis Healthcare seeking behaviour The likelihood to be prescribed with antibiotics by doctors (oral, intravenous or both) Combined behaviour score	13 14 18–20 32 40 41 45 47 48 50 51 76
Gender	The gender of the participant or caregiver	Self-medication with antibiotics Storing antibiotics at home Taking antibiotics as prophylaxis Healthcare seeking behaviour Combined behaviour score	13–15 19 20 28 35 45 48–51
Education	The education level of the participant, his/her parent or the caregiver	Asking/pressuring doctors for antibiotics Self-medication with antibiotics Storing antibiotics at home Over-the-counter purchases Taking antibiotics as prophylaxis Healthcare seeking behaviour Combined behaviour score	13 14 18–20 28 29 38 42 45 49–51 63 76
Income	The household income or monthly allowance of the participant or caregiver	Self-medication with antibiotics Storing antibiotics at home Taking antibiotics as prophylaxis	20 29 32 40 45 50 51
Location	The rural/urban of residence of the participant or caregiver	Asking/pressuring doctors for antibiotics Self-medication with antibiotics Over-the-counter purchases Storing antibiotics at home Taking antibiotics as prophylaxis The likelihood to be prescribed with antibiotics by doctors Combined behaviour score	13 18–20 24 29 43–45 47 49 50
Region	Region of residence of the participant or caregiver—geographic area or economic development stage	Asking/pressuring doctors for antibiotics Self-medication with antibiotics Over-the-counter purchases Storing antibiotics at home Taking antibiotics as prophylaxis The likelihood to be prescribed with antibiotics by doctors	13 20 28 50 51
Policy	Health policy or AMR programme that might affect prescribing or access to antibiotics (eg, measures to de-incentivise over-prescription in public health facilities, including decoupling the link between facility income and the sale of medicines and policy that bans over-the-counter purchases) Financial incentives for antibiotic prescribing of doctors	Self-medication with antibiotics Over-the-counter purchases* The likelihood to be prescribed with antibiotics by doctors	23 24 28
Norm	Participants’ view of how others treat illnesses and/or use antibiotics (non-China and non-predictor)* Healthcare providers reviewing others’ prescriptions (non-predictor)*	The likelihood to be prescribed with antibiotics by doctors*	59 75 77–79
Point-of-care	Prescribing habits/capacity might vary at different levels of health facilities: tertiary hospital, secondary/county hospital, community health centres/township hospital or private clinics/village clinics	No evidence available to date	–

*Non-predictor: effect is implied.

AMR, antimicrobial resistance; URTI, upper respiratory tract infections.

### Quantitative synthesis of factors influencing antibiotic use in the community

**Figure 2:**
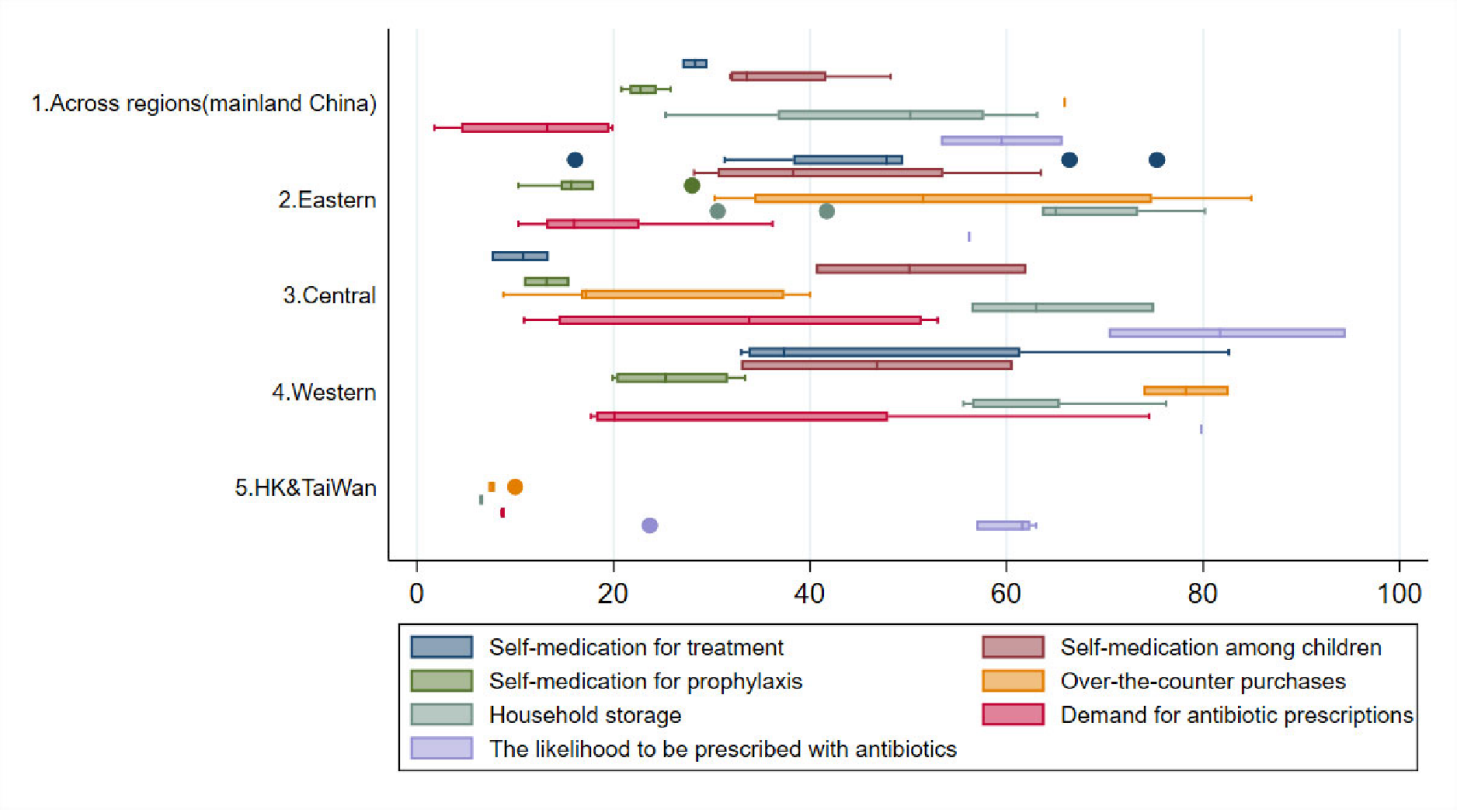
Synthesis of quantitative data on public antibiotic misuse behaviours in the community by study region.

In online supplemental file S3.1–3.22, we summarised the identified factors of antibiotic use, measures (eg, denominator, numerator and recall period) and geographic distributions of antibiotic use practices that have been studied across China. We found inconsistency in defining and measuring various types of antibiotic use behaviours, which raises issues of cross-study comparability and evaluation. A total of 49 studies quantitatively investigated factors influencing inappropriate antibiotic use either by patients, caregivers or providers within and beyond clinical settings. The synthesis of quantitative data on public antibiotic misuse behaviours in the community by study region is presented in figure 2.

#### Clinical settings

##### Antibiotic prescriptions for presumed self-limiting illnesses

The likelihood of being prescribed with antibiotics for presumed self-limiting illnesses during an outpatient clinic visit varied from 53.3%^13^ to 94.5%^14^ in mainland China and 23.7%^15^ to 63.0%^16^ in Hong Kong. Three studies found that 31.7%^17^ to about 50%^18^ of participants prescribed with antibiotics were administered them through intravenous infusion.^17–19^ Six studies investigated how patients’ socioeconomic backgrounds might influence antibiotic prescribing^13 14 17 20–22^ and among them, two identified patients’ antibiotics-related knowledge as a determinant.^14 17^ People with a medical background were less likely to receive antibiotic prescriptions and more likely to approve of that decision.^20^ Regional differences were also noted: living in regions of lower economic development was associated with an increased risk of antibiotic prescriptions for self-limiting illnesses.^13 20^ Three experiments using SPM were conducted in the past decade to investigate drivers of antibiotic misuse by providers and concluded that antibiotic dispensing practices in mainland China have been mainly influenced by financial incentives for prescriers and/or dispensing facilities,^23 24^ lack of diagnostic capacity^25^ and concerns over complications.^25–27^

##### Demand for antibiotic prescriptions

Demand for antibiotic prescriptions from patients and/or caregivers was reported in 21 studies, ranging from 1.8%^28^ to 74.5%^29^ in mainland China, compared with around 8.7%^16 21 30–32^ and 8.8%^33^ in Hong Kong and Taiwan, respectively. Out of the 21 studies, 9 identified factors influencing demands for antibiotic prescriptions.^13 17 19 20 29 30 34–36^ Two found knowledge to be a protective factor associated with demands for antibiotics.^17 36^ Having some level of medical education was found to have mixed effects.^20 37^ Older age,^13 19 20^ lower education levels^13 29^ and living in rural areas^29^ or regions with lower economic development^20^ were associated with an increased risk of inappropriate prescriptions by demand among patients and caregivers. People became less likely to demand antibiotic prescriptions after living in a region with better drug regulations.^38^ Two Hong Kong-based studies examined the reasons family doctors prescribed antibiotics for upper respiratory tract infections (URTI), and found reasons for this included ‘no energy to resist demand’,^26^ ‘lack of time’^26 39^ and ‘as a way to terminate the consultation’.^39^ They also found male doctors in Hong Kong to be more likely to over-prescribe antibiotics than their female peers.^26 39^

#### Community settings

##### Self-medication with antibiotics for treatment

The practice of self-medication with antibiotics for treatment was widely reported in 32 studies, all in mainland China. The overall prevalence of antibiotic self-medication (for therapeutic purposes) ranged from 7.6%^14^ to 82.6%^29^ in mainland China, with high prevalence found in Gansu (82.6%),^29^ Guangdong (63.5% in Guangzhou City),^40–42^ Shaanxi (60.6% in Xi’an City)^28 43 44^ and Jiangxi (62%).^18^ Out of 32 studies, 5 assessed the impact of knowledge on antibiotic self-medication with mixed results.^17 28 36 45 46^ Having an accepting attitude towards antibiotic self-medication^28^ and perceived susceptibility and perceived severity of the infection^28 47^ were associated with increased odds of antibiotic self-medication. Older age,^18 40 45 48^ being women^28 48 49^ and having more than one child in the house^18^ were associated with higher rates of antibiotic self-medication. The associations between antibiotic self-medication and education and urbanicity were inconsistent: some studies identified having higher education^18 29^ or living in the urban areas^43 44 49^ to be risk factors, while others came to the opposite conclusion.^18 28 29 42 45 49–51^ Having some level of medical education was associated with a higher likelihood of antibiotic self-medication compared with peers.^20 28 34 40 41 43 44 48 49 51^

##### Self-medication with antibiotics for prophylaxis

Patterns were similar for associations withself-medication with antibiotics as prophylaxis—often for URTI to prevent deterioration—measured in 11 studies,^13 17–20 36 38 43 50–52^ all in mainland China, with a prevalenceranging from 10.3%19 to 30.6%.^43^ Notably, regional differences were observedfor antibiotic self-medication, both for therapeutic purposes and prophylaxis:consistently, those living in highly economically developed regions were lesslikely to self-medicate with antibiotics, compared with their counterparts.^13 20 28 50 51^ Having health insurance was also associated with higher rates of antibiotic self-medication.^28^ Having the idea that antibiotics could preventhumans with a common cold from developing into more severe diseases wasassociated with backyard pig farmers adding antibiotics into pig feed.^52^

##### Over-the-counter (OTC) purchases of antibiotics

Access to non-prescription antibiotics, either via over-the-counter purchases or household storage, was strongly associated with antibiotic self-medication for therapeutic purposes^17 18 28 41 42 46 50 51^ or prophylaxis.^50 51^ The prevalence of over-the-counter (OTC) purchases of antibiotics ranged from 8.8%^14^ to 84.9%^52^ in mainland China, 7.3%^15 31^ to 7.8%^16 21 30 32^ in Hong Kong and was around 10.0%^33^ in Taiwan. Antibiotics were easily obtainable with very limited barriers from 48.5%^53^ to 83.6%^54^ of local pharmacies across mainland China when an acute diarrhoea or URTI was present in four experiments using SCM.^53-56^ The geographic location of the pharmacy,^53–55^ the distance from a hospital,^54^ being a chain pharmacy,^53^ having a special counter for antibiotics^54^ and having a licensed pharmacist on duty^53–55^ were all associated with OTC antibiotic dispensing. Backyard pig farmers who had purchased antibiotics for their pigs over the counter in the previous year were more likely to do so for humans.^52^

##### Household storage of antibiotics

The prevalence of household storage of antibiotics ranged from 25.3%^28^ to 80.2%^42^ in mainland China and was around 6% in Hong Kong,^16 21 31 32^ principally originating from over-the-counter purchases^17 41 50 51^ and leftover prescriptions.^16 17 31 41 50 51^ Being women,^13 19 20 50 51^ of older age,^13 50 51^ attaining higher education,^13 19 20 50 51^ having higher income^20 50 51^ and living in urban areas^19 20 50^ were associated with a higher likelihood of household storage of antibiotics. For backyard pig farmers, perceiving it good to store antibiotics at home was associated with higher risks for keeping antibiotics at home for both pigs and humans.^52^ Unsurprisingly, over-the-counter purchases^31^ were a risk factor for storing antibiotics at home.

### Qualitative studies

Factors of antibiotic use identified from nine qualitative and mixed methods studies^16 27 31 32 48 56–59^ generally supported the quantitative findings. Participants’ trust in their doctors^31^ made them not demand antibiotics; on the other hand, previous ‘successful’ experiences with similar symptoms prompted them to ask for antibiotics.^57^ Rural residents viewed self-medication, over-the-counter purchases for self-limiting conditions such as diarrhoea and colds, and storing antibiotics at home for future use, as norms.^58^ Inappropriate antibiotic dispensing was reported as a severe issue in less economically developed regions like Guizhou, where antibiotics became a routine prescription for patients suffering from any symptom other than fatigue, due to strong financial incentives for over-prescribing.^58^ From the prescribers’ perspective, lack of diagnostic capacity, such as inability to perform a routine blood test and a C reactive test, and fears of complications, such as pneumonia, bronchitis and otitis media, were the most frequently reported reasons for antibiotic prescriptions.^27^ Pressure to maintain a good doctor–patient relationship to maintain business was also reported as a reason to fulfil patients’ requests for antibiotic prescriptions.^27 59^ One study found clinical trainings to be effective in improving doctors’ knowledge and antibiotic prescribing behaviours.^59^ The overall context of pervasive non-prescription antibiotic dispensing in China can be attributed to the financial incentives from selling antibiotics and associated traditional Chinese medicines, inadequate Food and Drug Administration (FDA) supervision and lack of forceful regulations also prompted pharmacies to continue this practice for consumer retention.^56^

### Antibiotic use practices specific to the Chinese context

Among the 54 included studies, 11 studies^16–18 22 28 31 35 36 43 57 59^ found a misconception existed confusing anti-inflammatory medications and antibiotics, ranging in prevalence from 17.9%^17^ to 71.6%,^28^ even with 30.1% in paediatricians.^22^ Eleven studies^14 17 18 27 28 31 35 40 43 46 57^ reported a preference for intravenous injection of antibiotics, where 21.3%^17^–84.7%^46^ of participants believed infusion is much more efficacious than oral administration, and even 74% doctors thought the same way.^60^ In a less economically developed region like Guizhou, intravenous antibiotic treatment was common for mild diarrhoea, often in the absence of a proper diagnostic test.^58^ Mixing antibiotics with traditional Chinese medicine or preferences for traditional Chinese medicine over antibiotics for relieving cold symptoms were observed.^21 58^ One found users of traditional Chinese medicine were less likely to accept antibiotics when offered (OR=0.38, 95% CI 0.25 to 0.60) and were less likely to be treated with antibiotics for their last URTI (OR=0.49, 95% CI 0.27 to 0.81).^21^ Others found doctors prescribed antibiotics for URTI and combined antibiotic prescriptions with traditional Chinese medicine to relieve symptoms.^21 58^ Self-medication is common in the Chinese community; doctors reported their patients had self-medicated with antibiotics before reaching health facilities.^27 61^ Antibiotics were also widely used in animals by farmers without professional supervision.^52^

**Figure 3:**
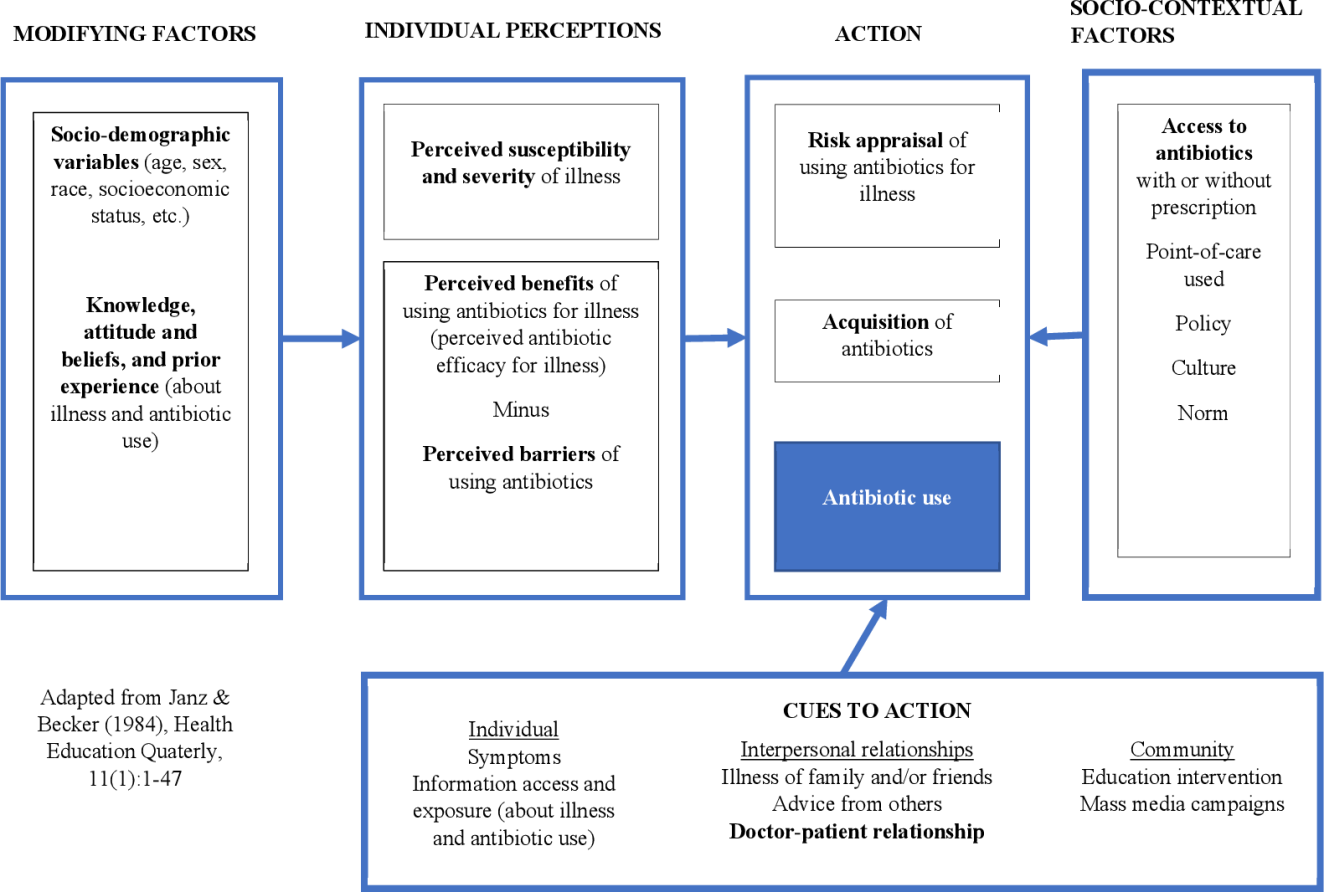
Modified health belief model for public antibiotic use.

The results of quality appraisal of the 54 studies were reported in online supplemental files S4–S6. Adapted from Health Belief Model, figure 3 presented a conceptual framework^9^ of non-biomedical factors that influence outpatient and community antibiotic use for common community-acquired infections.

## Discussion

### Summary of findings

In this systematic review, quantitative synthesis showed that inappropriate antibiotic use is pervasive throughout mainland China, given the relatively easy access to antibiotics, with or without a prescription. Access to non-prescription antibiotics, either via over-the-counter purchases or household storage, was strongly associated with antibiotic self-medication.^17 18 28 41 42 46 50 51^ Public AMR awareness levels were frequently measured to be high in mainland China^15–19 21 28 30 31 34 35 40 43 46–48 62 63^; however, there is little evidence that high awareness in China could lead to better antibiotic use. Striking regional differences were observed for antibiotic self-medication; those living in less economically developed regions were more likely to use antibiotics inappropriately.^13 20 28 50 51^ Having the experiences of OTC purchases for pig use was associated with a higher risk of obtaining antibiotics over the counter for human use in backyard pig farmers.^52^ Both quantitative and qualitative studies in this review revealed that doctor-patient relationships are critical in influencing unnecessary or inappropriate antibiotic prescriptions. Patients who trust their doctors, as well as people with some medical education or a higher education level would likely accept non-antibiotic prescriptions.^20 29 31^ Financial incentives for doctors led to inappropriate over-prescription of antibiotics.^23 24^ Non-prescription antibiotic sales were prevalent in community pharmacies.^53–56^ Antibiotic use is influenced by the local context in mainland China, where a misconception confusing anti-inflammatory medications and antibiotics,^16–18 28 31 35 36 43 57^ and a preference for intravenous injection of antibiotics^14 17 18 27 28 31 35 40 43 46 57^ are prevalent.

### Strengths and limitations of the review

To the best of our knowledge, this is the first mixed-methods systematic review of the prevalence, measures and factors of antibiotic use across China. This comprehensive review included studies across different regions of mainland China, Hong Kong and Taiwan, published in English and Chinese. It captured statistically assessed factors of actual antibiotic use behaviours by both healthcare providers and consumers, rather than only considering their knowledge, attitudes or intentions in isolation of these influencing factors. We further synthesised the findings using the Health Belief Model, which could identify a critical knowledge gap with a lack of evidence on several key factors on antibiotic use practice (see table 2) and inform the development of future behavioural change interventions to reduce antibiotic use in the community. The data and study design presented in the Chinese language publications were lean in general and therefore, for our review, we limited the inclusion to studies that had demonstrated sufficient rigour and detail in their reporting for us to appraise their evidence.

### Interpretation

Inappropriate use of antibiotics is influenced by non-biomedical factors within and beyond clinical settings that are unique to mainland China, yet common among low-income and middle-income countries, including public misconceptions,^16–18 22 28 31 35 36 43 57 59^ habitual use without professional guidance,^58^ insufficient FDA monitoring,^56^ incentivising the healthcare system towards prescribing and selling,^23 24 56^ lack of diagnostic capacity,^25 27^ and the delicate relationships between patients and prescribers,^27^ but some critical factors such as antibiotic literacy remained as research gaps. To date, there have been only few interventions implemented in primary care settings to reduce inappropriate prescribing,^64–70^ largely targeting clinicians and ignoring demand-side factors. Further, more research is needed to investigate the associations between human and animal use of antibiotics in rural China, where 564 million people reside^71^ in order to inform effective One Health interventions.

This study found an urgent need to take an evidence-based approach to identify determinants of antibiotic use practices within the target context, programme parameters for improvement and intervention components to optimise the use of antibiotics. These insights will be critical to tailor contextualised, multifaceted interventions for reducing inappropriate antibiotic use. For example, despite the AMR awareness campaigns invested in by the Chinese government, the inappropriate use of antibiotics was found to be prevalent across the country. Moreover, a study reported that well-intentioned government publicity about antibiotic abuse may have had the unintended consequence of increasing antibiotic prescriptions and exacerbating resistance.^58^ Such a phenomenon might be explained by the non-rational strategies people lean on while managing the type of risk and uncertainty associated with an acute infection: so-called tacit or experiential knowledge such as trust, intuition, emotion and prior ‘successful’ experiences with similar symptoms for healthcare decision making.^57 72^ Also, we found the national ban on over-the-counter purchases of antibiotics has been very limited in its impact—non-prescription purchases and use of antibiotics were reported across mainland China. Furthermore, few studies investigated the common practice—very much influenced by local context—in which physicians and pharmacists prepare cocktails of various medications, including traditional Chinese medicine and antibiotic agents for patients with URTI.^21 56 58^ Inappropriate antibiotic consumption is unlikely to decrease without multifaceted, context-tailored strategies targeting patients, prescribers and healthcare systems.

## Conclusion

This review revealed the impact of non-biomedical factors at individual, community, health system and societal levels on outpatient and community antibiotic use by healthcare users and providers in the Chinese context and demonstrated that they impact each other in an interactive manner. Given the large population size and consumption volume, the threat to human health from the adverse side effects of inappropriate use and drug resistance calls for immediate action. This study calls for multifaceted antibiotic stewardship programmes and strictive antibiotic drug policy, with a One Health framework. Future AMR strategies should incorporate an evidence-based, context-tailored design that simultaneously addresses drivers of antibiotic misuse from both the supply-side and demand-side within and beyond clinical settings.
