# Supplementary material for: Factors influencing inappropriate use of antibiotics in outpatient and community settings in China: a mixed-methods systematic review
Source: BMJ Glob Health. 2020 Nov 12;5(11):e003599. doi: 10.1136/bmjgh-2020-003599 (PMC7662435; doi:10.1136/bmjgh-2020-003599)
Supplement: Supplementary data [file bmjgh-2020-003599supp001.pdf]

S1. Search Criteria

Database	Search Strategy
MEDLINE	(("antimicrobial resistance"[tiab] OR "antibiotic*"[tiab] OR "Anti-Bacterial Agents/therapeutic use*"[Mesh] OR "Drug Resistance, Bacterial*"[Mesh]) AND ("behaviour*"[tiab] OR "use*"[tiab] OR "misuse"[tiab] OR "abuse"[tiab] OR "practice"[tiab] OR "consumption"[tiab] OR "supply"[tiab] OR "prescribing*"[tiab] OR "prescription*"[tiab] OR "prescribe*"[tiab] OR "utilization*"[tiab] OR "Practice Patterns, Physicians*"[Mesh] OR "Health Knowledge, Attitudes, Practice*"[mesh]) AND ("China"[tiab] OR "Hong Kong"[tiab] OR "Macau"[tiab] OR "Taiwan"[tiab]) AND ("1920/09/01"[EDAT] : "2020/05/31"[EDAT]) AND ("1920/09/01"[PDAT] : "2020/05/31"[PDAT]))
EMBASE	('awareness'/exp OR 'attitude to health'/exp OR 'clinical practice'/exp OR 'prescribe*':ab,ti OR 'prescription*':ab,ti OR 'prescribing*':ab,ti OR 'misuse':ab,ti OR 'abuse':ab,ti OR 'utilization*':ab,ti OR 'consumption*':ab,ti OR 'practice*':ab,ti) AND ('antibiotic agent'/exp OR 'antibiotic*':ab,ti) AND ('China':ab,ti OR 'Hong Kong':ab,ti OR 'Macau':ab,ti OR 'Taiwan':ab,ti) AND [1-1-1920]/sd NOT [31-05-2020]/sd NOT [animals]/lim NOT [medline]/lim
PsycINFO	(MA behaviour and behaviour mechanisms OR MA choice behaviour OR MA health knowledge, attitudes, practice* OR MA Practice Patterns, Physicians' OR AB usage OR AB use OR AB consum* OR AB behaviour* OR AB behaviour* OR AB "practice*") (AB AMR OR AB antimicrobial resistance OR AB antibiotic*) NOT ((animal* OR AB surgery OR AB Surgical OR AB dental OR AB cancer* OR AB Chronic obstructive pulmonary disease OR AB COPD OR AB alcohol OR AB tobacco OR AB addiction OR AB depression OR AB disorder* OR AB adherence OR AB diabet* OR MA Inpatients* OR AB inpatient* OR MA Hospitals OR AB tertiary OR AB HIV OR AB tuberculosis OR emergency[ti] OR ED[tiab] OR MA Intensive Care Units OR MA Economics OR AB analgesic* OR MA Hospitalization OR MA Health Care Facilities OR MA Health Care Facilities OR MA Patient Care Management)) NOT PO animal)) AND (AB China OR AB Taiwan OR AB hong kong OR AB macau)
CNKI (China National Knowledge Infrastructure)	((((题名=(抗生素+抗菌素+消炎药)) OR (Title=(抗生素+抗菌素+消炎药))) AND ((题名=(行为+使用)) OR (Title=(行为+使用)))) AND ((((摘要=(抗生素+抗生素滥用+抗生素使用+抗菌素+消炎药)) OR (ABSTRACT=(抗生素+抗生素滥用+抗生素使用+抗菌素+消炎药))) OR ((主题=(抗生素+抗生素滥用+抗生素使用+抗菌素+消炎药)) OR (题名=(抗生素+抗生素滥用+抗生素使用+抗菌素+消炎药)) OR (v_subject=(抗生素+抗生素滥用+抗生素使用+抗菌素+消炎药)))) AND (((摘要=(行为+自我治疗+自我药疗+无处方购买+处方)) OR (ABSTRACT=(行为+自我治疗+自我药疗+无处方购买+处方))) OR ((主题=(行为+自我治疗+自我药疗+无处方购买+处方)) OR (题名=(行为+自我治疗+自我药疗+无处方购买+处方)) OR (v_subject=(行为+自我治疗+自我药疗+无处方购买+处方)))))) 并且 发表时间 between (1900-1-1,2020-05-31) (精确匹配),专辑导航：医药卫生科技,社会科学II辑; 数据库：文献 跨库检索

1

Google Scholar	"antibiotics" and "China"

2

S2. Inclusion/Exclusion Criteria

	Inclusion Criteria	Exclusion
**Language**	English and Chinese	Other language
**Time period**	Inception of databases to June 2020	Everything else
**Population**	General public, caregivers of children (age < 18 years old), outpatients, adults; family doctors, physicians, clinicians, pharmacists, health care workers	Inpatients, animal only
**Study setting**	Community (primary care or outpatient setting)	Inpatient setting, Emergency Department
**Illness**	No restrictions	None
**Outcome**	Determinants of human antibiotic use behaviours (including self-medication with antibiotics or antibiotic prescribing, etc.)	Results of prescription analysis;<br>antibiotic knowledge or attitudes;<br>determinants of general self-medication, antibiotics not specifically mentioned;<br>for quantitative studies, those not presenting significant association between determinants and antibiotic use behaviours by multiple logistic regression analysis.
**Study design**	Empirical studies with original data (quantitative, qualitative and mix-methods)	Editorials, commentaries, reviews or literature reviews, descriptive studies, poster abstracts
**Others**		Low-quality studies with obvious data mistakes (e.g. data in figures or tables were different with it in context);<br>full-text article cannot be downloaded;<br>other non-relevant studies.

3

S3.1. Factors associated with self-medication with antibiotics without a prescription (East)

(Chronological order, the list starts with the most recently published studies)

Reference	Setting	Region	Last year of data collection	Study design	Non-biomedical factors	Description of the factor influencing outpatient and/or community antibiotic use	ORs
Zhu X, et al. 2016	Outpatient	East	N/A	Quantitative	Having some level of medical education	Prior knowledge of antibiotics	2.26 (1.59-3.22)
					Socio-Contextual (demographic characteristics)	Female gender	1.44 (1.01-2.05)
						Older age	1.25 (1.12-1.38)
Li Y, et al. 2016	Outpatient	East	N/A	Quantitative	Knowledge (combined knowledge score)	Combined knowledge score<br>Squared combined knowledge score	1.257 (1.081-1.461)<br>0.978 (0.963-0.992)
					Perceived barrier (or access) to antibiotics	Keeping antibiotics at home<br>Never<br>Often	 Reference<br>3.759 (2.759-5.122)
					Perceived Susceptibility	Self-rated health status<br>Poor<br>Good	 Reference<br>0.779 (0.620-0.978)
					Socio-Contextual (demographic characteristics)	Married<br>Not married	Reference<br>0.600 (0.476-0.757)
Tian L, et al. 2015	Outpatient	East	N/A	Quantitative	Having some level of medical education	Not majoring in medicine	2.746 (1.377-5.474)
					Socio-Contextual (demographic characteristics)	Female	2.542 (1.426-4.532)
					Socio-Contextual (socio-economic status)	Education:<br>Lower grade	3.550 (1.571-8.023)
						Hometown: Urban	2.186 (1.129-4.231)
Liao R et al, 2013	Outpatient	East	N/A	Quantitative	Having some level of medical education	Having caregivers working in the medical field	1.744 (1.107-2.746)
					Perceived barrier (or access) to antibiotics	Keeping antibiotics at home	1.529 (1.169-2.001)
					Socio-Contextual (demographic characteristics)	Parents' age	0.807 (0.680-0.957)
Yao Z et al, 2013	Outpatient	East	N/A	Quantitative	Perceived barrier (or access) to antibiotics	Keeping antibiotics at home	4.792 (3.541-6.485)
					Socio-Contextual (socio-economic status)	Education:<br>Postgraduate and above<br>Elementary school and below<br>Junior high school<br>High school or secondary school	 Reference<br>5.042 (1.495-17.002)<br>2.358 (1.150-4.838)<br>2.104 (1.106-4.003)
Huang Y, et al. 2013	Outpatient	East	N/A	Quantitative	Having some level of medical education	Medical students vs Non-medical students	OR not applicable
Pan H, et al. 2012	Outpatient	East	N/A	Quantitative	Having some level of medical education	Prior knowledge of antibiotics	2.23 (1.74-2.87)
					Socio-Contextual (demographic characteristics)	Age	1.23 (1.16-1.30)
					Socio-Contextual (socio-economic status)	Higher allowance:<br>≤500 RMB<br>500 to 1,000 RMB<br>>1000 RMB	 Reference<br>1.49 (1.17-1.91)<br>2.18 (1.29-3.68)

S3.2. Factors associated with self-medication with antibiotics without a prescription (Central)

Reference	Setting	Region	Last year of data collection	Study design	Non-biomedical factors	Description of the factor influencing outpatient and/or community antibiotic use	ORs
Yu M, et al. 2014	Outpatient	Central	N/A	Quantitative	Perceived barrier (or access) to antibiotics	Once purchased antibiotics without physicians' prescription	6.264 (4.144-9.469)
						Sometimes, often or always stores antibiotics at home	2.792 (1.961-3.975)
					Antibiotic use behaviors	Would follow all the advice from physicians	0.639 (0.451-0.906)
					Socio-Contextual (demographic characteristics)	Raising more than one child	2.174 (1.485-3.183)
						Age of children	1.146 (1.037-1.266)
					Socio-Contextual (socio-economic status)	Education:<br>College or above<br>Primary school	 Reference<br>0.191 (0.049-0.754)
						Living in villages	1.643 (1.108-2.436)

S3.3. Factors associated with self-medication with antibiotics without a prescription (West)

Reference	Setting	Region	Last year of data collection	Study design	Non-biomedical factors	Description of the factor influencing outpatient and/or community antibiotic use	ORs
Lv B, et al. 2014.	Outpatient	West	N/A	Quantitative	Having some level of medical education	Medical students	1.612 (1.193–2.178)
					Socio-Contextual (region/location)	From urban areas	1.495 (1.103–2.026)
Lv B, 2013	Outpatient	West	N/A	Quantitative	Having some level of medical education	Majoring in medicine	1.697 (1.229-2.341)
					Socio-Contextual (socio-economic status)	Hometown:<br>Urban	1.527 (1.109-2.203)
Jin Y, et al. 2014	Outpatient	West	N/A	Quantitative	Socio-Contextual (socio-economic status)<br>Behaviour: over-the-counter purchase and use of antibiotics according to commercial advertisement	Hometown:<br>Rural<br>Urban	Reference<br>0.71 (0.60-0.84)
						Education:<br>Senior high school and above<br>Junior high school and below	Reference<br>1.72 (1.45-2.03)
					Socio-Contextual (socio-economic status)<br>Behaviour: over-the-counter purchase and use of antibiotics	Hometown:<br>Rural<br>Urban	Reference<br>0.56 (0.49-0.66)
						Education:<br>Senior high school and above<br>Junior high school and below	Reference<br>0.70 (0.60-0.81)

S3.4. Factors associated with self-medication with antibiotics without a prescription (across regions)

Reference	Setting	Region	Last year of data collection	Study design	Non-biomedical factors	Description of the factor influencing outpatient and/or community antibiotic use	ORs
Wang W, et al. 2019	Outpatient	Across regions	N/A	Quantitative	Knowledge (misconceptions)	Having the misconception that antibiotic is a Xiaoyanyao	1.51 (1.21-1.89)
Chang J, et al. 2018	Outpatient	Across regions	N/A	Quantitative	Perceived barrier (or access) to antibiotics	Knowing prescription-only regulation for sales of antibiotics at community pharmacies	0.77 (0.66-0.91)
					Attitudes (accepting attitudes towards SMA)	Caregivers' supportive attitude	2.66 (2.21-3.19)
					Having some level of medical education	Having family member or relatives working in health sector	1.38 (1.14-1.66)
					Perceived barrier (or access) to antibiotics	Keeping antibiotics at home	6.25 (4.73-8.26)
					Perceived Susceptibility	Child's rated health status rated:<br>~~F as f~~air, poor, or very poor<br>Good or very good	Reference<br>0.48 (0.40-0.57)
					Socio-Contextual (demographic characteristics)	Caregiver's relationship with children was grandparents	0.68 (0.49-0.94)
						Caregiver's gender: Female	1.25 (1.06–1.47)
					Socio-Contextual (socio-economic status)	Caregivers with senior high school or equivalent	0.75 (0.57-0.98)
					Socio-Contextual (region/location)	Xi'an<br>Shanghai<br>Changsha	Reference<br>0.34 (0.28-0.42)<br>0.78 (0.65-0.94)
					Perceived barrier (or access) to antibiotics/healthcare	Having children's health insurance	1.30 (1.05-1.61)
Peng D, et al. 2018	Outpatient	Across regions	N/A	Quantitative	Having some level of medical education	Parent's having medical background	3.01 (1.66-5.47)
					Socio-Contextual (region/location)	Zhejiang<br>Guizhou	Reference<br>3.00 (1.84-4.90)
Wang X, et al. 2019	Outpatient	Across regions	N/A	Quantitative	Perceived barrier (or access) to antibiotics (over-the-counter purchase, keeping antibiotics at home, leftover prescriptions)	Keep Antibiotics at Home:<br>No<br>Yes (Previously bought from pharmacies)<br>Yes (Previously prescribed by doctors)<br>Yes (Other)	Reference<br>5.29 (3.72-7.53)<br>4.03 (2.68-6.07)<br>6.06 (3.06-12.02)
					Socio-Contextual (socio-economic status)	Urban	0.65 (0.49-0.88)
					Socio-Contextual (region/location)	Zhejiang University<br>Lanzhou University<br>Jilin University<br>Nankai University<br>Guizhou University	Reference<br>2.38 (1.55-3.65)<br>3.07 (1.99-4.71)<br>2.37 (1.52-3.69)<br>2.27 (1.44-3.58)
Wang X, et al. 2017	Outpatient	Across regions	N/A	Quantitative	Knowledge (combined knowledge score)	0-4<br>5-9<br>10-13	Reference<br>0.53 (0.39-0.72)<br>0.36 (0.24-0.54)
					Perceived barrier (or access) to antibiotics	Keeping antibiotics at home	5.05 (3.58-7.14)
Li R, et al 2016	Outpatient	Across regions	N/A	Quantitative	Knowledge<br>Socio-Contextual (demographic characteristics)	Guardians having basic health knowledge	0.82 (0.79-0.86)
						Female children	0.92 (0.88-0.96)
						Being raised by parents	0.90 (0.85-0.94)
						Children's age:<br>1–3 years<br>4–6 years	 1.62 (1.54-1.71)<br>1.90 (1.77-2.03)
						Higher education of guardians	0.60 (0.55-0.66)
						Western China:<br>Low income<br>Middle income<br>Higher income<br>Eastern China:<br>Low income<br>Higher income<br>Middle China:<br>Low income<br>Middle income	 Reference<br>1.63 (1.51-1.78)<br>1.71 (1.50-1.94)<br><br>Reference<br>0.75 (0.65-0.86)<br><br>Reference<br>0.86 (0.77-0.96)
						Urban area	0.79 (0.76-0.83)
Sun C, et al. 2019	Outpatient	Across regions	N/A	Quantitative	Having some level of medical education	Parents with medical background	0.71 (0.52-0.97)
					Perceived barrier (or access) to antibiotics (over-the-counter purchase, keeping antibiotics at home, leftover prescriptions)	Parents who did not keep antibiotics at home<br>Parents who had leftover antibiotics<br>1) which came from previous prescriptions<br>2) which were purchased	Reference<br>3.80 (2.89-5.00)<br>6.45 (4.89-8.51)

Formatted: Font: (Asian) Chinese (PRC)

						from pharmacies	
						Parents who kept antibiotics at home for children	4.91 (3.84-6.28)
					Socio-Contextual (socio-economic status)	Parents' highest level of education:<br>Middle school and below<br>College and above	Reference<br>0.72 (0.52-0.99)
						Average household income (RMB, monthly)<br><3000 (US$461)<br>5001–10,000 (US$770–1538):	Reference<br>0.65 (0.46-0.91)
					Socio-Contextual (region/location)	Province:<br>Zhejiang<br>Guangxi<br>Shaanxi	Reference<br>1.91 (1.38-2.65)<br>2.63 (1.91-3.60)
Xu Y, et al. 2020	Outpatient	Across regions	N/A	Quantitative	Socio-Contextual (region/location)	Province:<br>Zhejiang<br>Shaanxi	Reference<br>2.82 (2.06-3.86)

S3.5. Factors associated with taking antibiotics as prophylaxis (East)

Reference	Setting	Region	Last year of data collection	Study design	Non-biomedical factors	Description of the factor influencing outpatient and/or community antibiotic use	ORs
Dyar O. J., et al. 2018	Outpatient	East	N/A	Quantitative	Perceived benefits and disbenefits (antibiotic efficacy)	Backyard pig farmers who would only use antibiotics when their pigs have signs of disease<br>Backyard pig farmers who think that taking antibiotics can prevent a common cold in humans developing into a more severe disease such as pneumonia<br>Behaviour outcome: always or often adding antibiotics into pig feed	32%<br>52%<br>p<0.001*

* The authors employed a stratified analysis.

S3.6. Factors associated with taking antibiotics as prophylaxis (across regions)

Reference	Setting	Region	Last year of data collection	Study design	Non-biomedical factors	Description of the factor influencing outpatient and/or community antibiotic use	ORs
Wang W, et al. 2019	Outpatient	Across regions	N/A	Quantitative	Knowledge (misconceptions)	Having the misconception that antibiotic is a Xiaoyanyao	1.36 (1.24-1.50)
Hu Y, et al. 2018	Outpatient	Across regions	N/A	Quantitative	Socio-Contextual (socio-economic status)	Hometowns were urban	0.69 (0.50-0.94)
Peng D, et al. 2018	Outpatient	Across regions	N/A	Quantitative	Having some level of medical education	Majoring in medicine	0.69 (0.55-0.87)
						Parent's having medical background	1.45 (1.08-1.95)
					Socio-Contextual (region/location)	Zhejiang<br>Guizhou	Reference<br>2.28 (1.89-2.76)
Wang X, et al. 2018	Outpatient	Across regions	N/A	Quantitative	Having some level of medical education	Majoring in medicine	0.52 (0.44-0.60)
						Parent with Medical Background	1.47 (1.26-1.72)
					Perceived barrier (or access) to antibiotics (over-the-counter purchase, keeping antibiotics at home, leftover prescriptions)	Keep Antibiotics at Home:<br>No<br>Yes (Previously bought from pharmacies)<br>Yes (Previously prescribed by doctors)<br>Yes (Other)	Reference<br>2.55 (2.22-2.92)<br>2.62 (2.34-2.93)<br>2.72 (1.97-3.76)
					Socio-Contextual (socio-economic status)	Household Income:<br><3000 ($461 USD)<br>3000-10,000 ($462-$1538 USD)	Reference<br>0.88 (0.79-0.99)
						Hometowns were urban	0.80 (0.71-0.90)
					Socio-Contextual (region/location)	Zhejiang University<br>Lanzhou University<br>Jilin University<br>Guizhou University	Reference<br>1.87 (1.58-2.22)<br>1.99 (1.69-2.35)<br>2.18 (1.83-2.58)
Wang X, et al. 2017	Outpatient	Across regions	N/A	Quantitative	Knowledge (combined knowledge score)	0-4<br>5-9<br>10-13	Reference<br>0.64 (0.57-0.72)<br>0.35 (0.30-0.41)
Lu J, et al. 2019	Outpatient	Across regions	N/A	Quantitative	Socio-Contextual (socio-economic status)	University students stayed in Eastern China<br>University students moved to Western China from Eastern China	Reference<br>1.85 (1.32-2.56)
						University students stayed in Western China<br>University students moved to Eastern China from Western China	Reference<br>0.57 (0.41-0.81)
						Specialty (among university students from Eastern China or attended Eastern universities):<br>Social Science<br>Science	Reference<br>1.55 (1.16-2.08)
						Specialty (among university students from Western or attended Western universities):<br>Social Science<br>Science	Reference<br>1.44 (1.16-1.78)
Sun C, et al. 2019	Outpatient	Across regions	N/A	Quantitative	Having some level of medical education	Parents with medical background	0.62 (0.52-0.75)
					Perceived barrier (or access) to antibiotics (over-the-counter purchase, keeping antibiotics at home, leftover prescriptions)	Parents who did not keep antibiotics at home<br>Parents who had leftover antibiotics<br>1) which came from previous prescriptions<br>2) which were purchased from pharmacies	Reference<br>2.96 (2.62-3.34) 3.53 (3.07-4.05)

						Parents who kept antibiotics at home for children	3.16 (2.83-3.53)
					Socio-Contextual (demographic characteristics)	Sex of caregiver:<br>Male<br>Female	Reference<br>0.83 (0.74-0.94)
					Socio-Contextual (socio-economic status)	Parents' highest level of education:<br>Middle school and below<br>College and above	Reference<br>0.85 (0.73-0.99)
					Socio-Contextual (region/location)	Province:<br>Zhejiang<br>Shaanxi	Reference<br>1.59 (1.38-1.84)
Xu Y, et al. 2020	Outpatient	Across regions	N/A	Quantitative	Having some level of medical education	Parents' medical background:<br>No<br>Yes	Reference<br>0.62 (0.50-0.77)
					Socio-Contextual (demographic characteristics)	Age of child	1.02 (1.01-1.04)
					Socio-Contextual (region/location)	Province:<br>Zhejiang<br>Shaanxi	Reference<br>1.82 (1.58-2.10)

S3.7. Factors associated with over-the-counter purchase of antibiotics (East)

Reference	Setting	Region	Last year of data collection	Study design	Non-biomedical factors	Description of the factor influencing outpatient and/or community antibiotic use	ORs
Dyar O. J., et al. 2018	Outpatient	East	N/A	Quantitative	Perceived barriers/access to antibiotics (over-the-counter-purchase)	Backyard pig farmers reported that they hadn’t bought antibiotics for their pigs without first speaking with a vet in the previous year<br>Backyard pig farmers reported that they had bought antibiotics for their pigs without first speaking with a vet in the previous year	25%<br>49%<br>p<0.001*

* The authors employed a stratified analysis.

S3.8. Factors associated with over-the-counter purchase of antibiotics (Central)

Reference	Setting	Region	Last year of data collection	Study design	Non-biomedical factors	Description of the factor influencing outpatient and/or community antibiotic use	ORs
Wang J, et al. 2017	Outpatient	Central	N/A	Quantitative	Prior experience	Whether been prescribed antibiotics by doctors or not	β =0.239*
					Socio-Contextual (demographic characteristics)	Age of child	β =-0.074*
					Socio-Contextual (socio-economic status)	Parents' education level	β =0.090*

* The authors employed linear regression analyses.

S3.9. Factors associated with over-the-counter purchase of antibiotics (West)

Reference	Setting	Region	Last year of data collection	Study design	Non-biomedical factors	Description of the factor influencing outpatient and/or community antibiotic use	ORs
Jin Y, et al. 2014	Outpatient	West	N/A	Quantitative	Socio-Contextual (socio-economic status)<br>Behaviour: over-the-counter purchase and use of antibiotics	Hometown:<br>Rural<br>Urban	Reference<br>0.56 (0.49-0.66)
						Education:<br>Senior high school and above<br>Junior high school and below	Reference<br>0.70 (0.60-0.81)
					Socio-Contextual (socio-economic status)<br>Behaviour: over-the-counter purchase and use of antibiotics according to commercial advertisements	Hometown:<br>Rural<br>Urban	Reference<br>0.71 (0.60-0.84)
						Education:<br>Senior high school and above<br>Junior high school and below	Reference<br>1.72 (1.45-2.03)

S3.10. Factors associated with over-the-counter purchase of antibiotics (across regions)

Reference	Setting	Region	Last year of data collection	Study design	Non-biomedical factors	Description of the factor influencing outpatient and/or community antibiotic use	ORs
Peng D, et al. 2018	Outpatient	Across regions	N/A	Quantitative	Having some level of medical education	Parent's having medical background	0.62 (0.43-0.89)
					Socio-Contextual (socio-economic status)	Education:<br>Undergraduate<br>Graduate	Reference<br>1.94 (1.35,2.80)
					Socio-Contextual (region/location)	Zhejiang<br>Guizhou	Reference<br>1.71(1.36,2.15)

S3.11. Factors associated with over-the-counter purchase of antibiotics (Hong Kong and Taiwan)

Reference	Setting	Region	Last year of data collection	Study design	Non-biomedical factors	Description of the factor influencing outpatient and/or community antibiotic use	ORs
Lam TP, et al. 2015	Outpatient	Hong Kong	N/A	Mixed-methods (qualitative + quantitative)	Socio-Contextual (socio-economic status)	Local-born<br>New immigrants<br>All immigrants	Reference<br>2.205 (1.230- 3.953)<br>0.601 (0.436-0.829)
Wun YT, et al. 2015	Outpatient	Hong Kong	N/A	Mixed-methods (qualitative + quantitative)	Socio-Contextual (socio-economic status)	Local-born<br>Recent-immigrants	Reference<br>2.37 (1.28-4.15)
Wun YT, et al. 2013	Outpatient	Hong Kong	N/A	Mixed-methods (qualitative + quantitative)	Socio-Contextual (demographic characteristics)	Age	Not reported
					Risk perception/Perceived severity	Those who agreed with the potential harm of such practice	0.47 (0.341-0.654)

16

S3.12. Factors associated with storing antibiotics at home (East)

Reference	Setting	Region	Last year of data collection	Study design	Non-biomedical factors	Description of the factor influencing outpatient and/or community antibiotic use	ORs
Dyar O. J., et al. 2018	Outpatient	East	N/A	Quantitative	Attitudes (ok to store/self-medicate with antibiotics)	Backyard pig farmers who did not think it was good to store leftover antibiotics for their pigs<br>Backyard pig farmers who thought it was good to store leftover antibiotics for their pigs<br>Behaviour outcome: have stored antibiotics for pigs	20%<br>41%<br>p<0.001<br>(stratified analysis)
						Backyard pig farmers who did not think it was good to store leftover antibiotics for their pigs<br>Backyard pig farmers who thought it was good to store leftover antibiotics for their pigs<br>Behaviour outcome: have stored antibiotics for humans	32%<br>47%<br>p<0.001<br>(stratified analysis)

S3.13. Factors associated with storing antibiotics at home (West)

Reference	Setting	Region	Last year of data collection	Study design	Non-biomedical factors	Description of the factor influencing outpatient and/or community antibiotic use	ORs
Jin Y, et al. 2014	Outpatient	West	N/A	Quantitative	Socio-Contextual (socio-economic status)	Hometown:<br>Rural<br>Urban	Reference<br>1.30 (1.06-1.58)
						Education:<br>Senior high school and above<br>Junior high school and below	Reference<br>1.31 (1.11-1.55)
						Household income (per month):<br>≥2000 RMB<br><1000 RMB<br>1000-1999 RMB	Reference<br>0.46 (0.36-0.60)<br>0.69 (0.55-0.88)

S3.14. Factors associated with storing antibiotics at home (across regions)

Reference	Setting	Region	Last year of data collection	Study design	Non-biomedical factors	Description of the factor influencing outpatient and/or community antibiotic use	ORs
Hu Y et al, 2018	Outpatient	Across regions	N/A	Quantitative	Having some level of medical education	Mothers with medical backgrounds:<br>Yes<br>No	Reference<br>0.53 (0.32-0.88)
					Socio-Contextual (demographic characteristics)	Female students	1.20 (1.04–1.56)
					Socio-Contextual (socio-economic status)	Fathers had a higher educational level	1.60 (1.10-2.30)
						Hometowns were urban	1.60 (1.20-1.90)
Peng D, et al. 2018	Outpatient	Across regions	N/A	Quantitative	Having some level of medical education	Parent's having medical background	1.68 (1.24-2.27)
					Socio-Contextual (demographic characteristics)	Males	0.70 (0.61-0.80)
					Socio-Contextual (socio-economic status)	Education level of parents:<br>Illiteracy/primary school<br>Junior high school<br>Senior high school<br>University/above	Reference<br>1.33 (1.07-1.66)<br>1.70 (1.32-2.17)<br>2.03 (1.53-2.69)
						Household income per month:<br>< 3000 RMB<br>3000-10,000 RMB	Reference<br>1.30 (1.10-1.53)
						Hometowns were rural	0.64 (0.54-0.76)
Wang X, et al. 2018	Outpatient	Across regions	N/A	Quantitative	Having some level of medical education	Parent with Medical Background	1.56 (1.33-1.84)
					Socio-Contextual (demographic characteristics)	Females	1.47 (1.35-1.59)
						Age	1.02 (1.00-1.04)
					Socio-Contextual (socio-economic status)	Household Income:<br><3000 ($461 USD)<br>3000-10,000 ($462-$1538 USD)	Reference<br>1.15 (1.04-1.27)
						Education level of parents:<br>Illiteracy/primary school<br>Junior high school<br>Senior high school<br>University/above	Reference<br>1.27 (1.09-1.47)<br>1.54 (1.32-1.81)<br>1.79 (1.51-2.13)
						Hometowns were urban	1.50 (1.35-1.66)
					Socio-Contextual (region/location)	Zhejiang University<br>Wuhan University	Reference<br>0.83 (0.72-0.96)
Wang X, et al. 2017	Outpatient	Across regions	N/A	Quantitative	Knowledge (combined knowledge score)	0-4<br>5-9	Reference<br>1.29 (1.15-1.45)
Sun C, et al. 2019	Outpatient	Across regions	N/A	Quantitative	Having some level of medical education	Respondents with medical backgrounds	1.54 (1.35-1.75)
					Socio-Contextual (demographic characteristics)	Fathers<br>Mothers	Reference<br>1.17 (1.06-1.29)
						Age of child	1.05 (1.04-1.07)
					Socio-Contextual (socio-economic status)	Higher education levels<br>Middle school and below<br>High school<br>College and above	Reference<br>1.34 (1.20-1.51)<br>1.50 (1.33-1.70)
						Average household income (RMB, monthly)<br><3000 (US$461)<br>3000–5000 (US$462–769)<br>5001–10 000 (US$770–1538)<br>>10 000 (US$1538)	Reference<br>1.22 (1.08-1.38)<br>1.17 (1.02-1.33)<br>1.36 (1.16-1.60)
					Socio-Contextual (region/location)	Province:<br>Zhejiang<br>Shaanxi	Reference<br>1.96 (1.75-2.20)
Xu Y, et al. 2020	Outpatient	Across regions	N/A	Quantitative	Having some level of medical education	Parent's medical background	1.60 (1.37-1.88)
					Socio-Contextual (demographic characteristics)	Age of the child	1.05 (1.03-1.06)
						Gender of the parent:<br>Male<br>Female	Reference<br>1.22 (1.08-1.39)
					Socio-Contextual (socio-economic status)	Parents' highest level of education:<br>Middle school or below<br>High school<br>College or above	Reference<br>1.38 (1.19-1.60)<br>1.55 (1.33-1.81)
						Residential location:<br>Urban<br>Rural	Reference<br>0.88 (0.78-0.98)
					Socio-Contextual (region/location)	Province:<br>Zhejiang<br>Shaanxi	Reference<br>1.93 (1.72-2.17)

Formatted: Font: (Asian) Chinese (PRC)

Formatted: Space After: 0 pt

S3.15. Factors associated with storing antibiotics at home (Hong Kong and Taiwan)

Reference	Setting	Region	Last year of data collection	Study design	Non-biomedical factors	Description of the factor influencing outpatient and/or community antibiotic use	ORs
Lam TP, et al. 2015	Outpatient	Hong Kong	N/A	Mixed-methods (qualitative + quantitative)	Socio-Contextual (socio-economic status)	Local-born<br>New immigrants	Reference<br>2.490 (1.385-4.477)
Wun YT, et al. 2015	Outpatient	Hong Kong	N/A	Mixed-methods (qualitative + quantitative)	Socio-Contextual (socio-economic status)	Local-born<br>Recent-immigrants	Reference<br>2.37 (1.29-4.15)
Wun YT et al. 2013	Outpatient	Hong Kong	N/A	Mixed-methods (qualitative + quantitative)	Socio-Contextual (demographic characteristics)	Age	Not reported
					Socio-Contextual (socio-economic status)	Income	Not reported

S3.16. Factors associated with asking/pressuring doctors for antibiotics (East)

**Reference**	**Setting**	**Region**	**Last year of data collection**	**Study design**	**Non-biomedical factors**	**Description of the factor influencing outpatient and/or community antibiotic use**	**ORs**
Huang Y, et al, 2013	Outpatient	East	N/A	Quantitative	Having some level of medical education	Medical students vs Non-medical students	OR not applicable

S3.17. Factors associated with asking/pressuring doctors for antibiotics (West)

Reference	Setting	Region	Last year of data collection	Study design	Non-biomedical factors	Description of the factor influencing outpatient and/or community antibiotic use	ORs
Jin Y, et al. 2014	Outpatient	West	N/A	Quantitative	Socio-Contextual (socio-economic status)<br>Behaviour: asking doctors for antibiotics	Hometown:<br>Rural<br>Urban	Reference<br>0.73 (0.63-0.85)
						Education:<br>Senior high school and above<br>Junior high school and below	Reference<br>1.18 (1.01-1.37)
					Socio-Contextual (socio-economic status)<br>Behaviour: asking doctors for antibiotics via intravenous injection	Hometown:<br>Rural<br>Urban	Reference<br>0.78 (0.67-0.91)

S3.18. Factors associated with asking/pressuring doctors for antibiotics (across regions)

Reference	Setting	Region	Last year of data collection	Study design	Non-biomedical factors	Description of the factor influencing outpatient and/or community antibiotic use	ORs
Wang W, et al. 2019	Outpatient	Across regions	N/A	Quantitative	Knowledge (misconceptions)	Having the misconception that antibiotic is a Xiaoyanyao	1.34 (1.21-1.48)
Hu Y et al, 2018	Outpatient	Across regions	N/A	Quantitative	Socio-Contextual (demographic characteristics)	Aged between 16 and 20 years old<br>Aged between 21 and 30 years old	Reference<br>1.50 (1.00-2.20)
Peng D, et al. 2018	Outpatient	Across regions	N/A	Quantitative	Having some level of medical education	Majoring in medicine	0.71 (0.56-0.90)
					Socio-Contextual (demographic characteristics)	Age	1.05 (1.00-1.11)
					Socio-Contextual (socio-economic status)	Education level of parents:<br>Illiteracy/primary school<br>Senior high school	Reference<br>1.39 (1.01-1.91)
					Socio-Contextual (region/location)	Zhejiang<br>Guizhou	Reference<br>1.48 (1.22-1.80)
Wang X, et al. 2017	Outpatient	Across regions	N/A	Quantitative	Knowledge (combined knowledge score)	0-4<br>5-9<br>10-13	Reference<br>0.71 (0.62-0.80)<br>0.50 (0.42-0.59)
Lu J, et al. 2019	Outpatient	Across regions	N/A	Quantitative	Socio-Contextual (socio-economic status)	University students stayed in Eastern China<br>University students moved to Western China from Eastern China	Reference<br>2.13 (1.54-3.03)
						University students stayed in Western China<br>University students moved to Eastern China from Western China	Reference<br>0.56 (0.37-0.83)
						Specialty (among university students from Eastern China or attended Eastern universities):<br>Social Science<br>Science	Reference<br>1.49 (1.09-2.04)
						Parent's education level (among university students from Western China or attended Western universities):<br>Middle School or Below College or Above	Reference<br>0.70 (0.49-0.99)
Xu Y, et al. 2020	Outpatient	Across regions	N/A	Quantitative	Socio-Contextual (demographic characteristics)	Age of the child	1.08 (1.02-1.14)
					Socio-Contextual (socio-economic status)	Parents' highest level of education<br>Middle school or below<br>High school<br>College or above	Reference<br>0.50 (0.29-0.86)<br>0.46 (0.27-0.78)

S3.19. Factors associated with antibiotic prescriptions (Central)

Outcome: likelihood of being prescribed with antibiotics

Reference	Setting	Region	Last year of data collection	Study design	Non-biomedical factors	Description of the factor influencing outpatient and/or community antibiotic use	ORs
Chai J et al. 2019	Outpatient	Central	N/A	Quantitative	Knowledge (combined knowledge score)	Zero<br>≥3 scores<br>Outcome: being prescribed oral/intravenous antimicrobials	reference<br>0.32 (0.13-0.78)
					Socio-Contextual (demographic characteristics)	Age<br>Outcome: being prescribed oral antimicrobial use	0.81 (0.71-0.93)
						Age<br>Outcome: being prescribed intravenous antimicrobial use	1.21 (1.10-1.33)

S3.20. Factors associated with antibiotic prescriptions (West)

Reasons for antibiotic prescription to children with upper respiratory tract infections

Reference	Setting	Region	Last year of data collection	Study design	Non-biomedical factors	Description of the factor influencing outpatient and/or community antibiotic use	ORs
Ye D, et al. 20	Outpatient	West	N/A	Quantitative	Perceived barriers/access to antibiotics (asking/pressuring doctors for antibiotics)	Always, n (%)<br>Often, n (%)<br>Sometimes, n (%)<br>Seldom, n (%)<br>Never, n (%)	7 (1.5)<br>50 (10.6)<br>138 (29.2)<br>154 (32.6)<br>123 (26.1)

S3.21. Factors associated with antibiotic prescriptions (across regions)

Outcome: likelihood of being prescribed with antibiotics

Reference	Setting	Region	Last year of data collection	Study design	Non-biomedical factors	Description of the factor influencing outpatient and/or community antibiotic use	ORs
Peng D et al, 2018	Outpatient	Across regions	N/A	Quantitative	Having some level of medical education	Majoring in medicine	0.49 (0.26-0.93)
					Socio-Contextual (socio-economic status)	Hometown were rural	2.01 (1.05-3.84)
					Socio-Contextual (region/location)	Province:<br>Zhejiang<br>Guizhou	Reference<br>2.95 (1.68-5.18)
Wang X, et al. 2017	Outpatient	Across regions	N/A	Quantitative	Knowledge (combined knowledge score)	0-4<br>5-9<br>10-13	Reference<br>0.58 (0.39-0.87)<br>0.46 (0.27-0.76)
Xu Y, et al. 2020	Outpatient	Across regions	N/A	Quantitative	Socio-Contextual (socio-economic status)	Residential location:<br>Urban<br>Rural	Reference<br>0.67 (0.54-0.82)
					Socio-Contextual (region/location)	Province<br>Zhejiang<br>Shaanxi	Reference<br>1.46 (1.16-1.84)

S3.22. Factors associated with antibiotic prescriptions (Hong Kong and Taiwan)

Reference	Setting	Region	Last year of data collection	Study design	Non-biomedical factors	Description of the factor influencing outpatient and/or community antibiotic use	ORs
Wun YT et al. 2014	Outpatient	Hong Kong	N/A	Quantitative	Health care seeking behaviors	TCM (Traditional Chinese medicine)-attenders	0.38 (0.25-0.60) for accepting antibiotics when offered
					Health care seeking behaviors		0.49 (0.27-0.81) for being treated with antibiotics in last URTI

S4. Appraisal – quantitative

Author	Region of China	Study design	Data collection method	Target population	Eligibility criteria (yes/no)	Sample size	Response rate (%)	Clarity of the questions/statements (yes/no)	Ethical considerations (yes/no)	Clarity of data (including numerators, denominators, and missing values) (yes/no)	Consistency between the research question and data reported (yes/no)
**Chan, Y. H., 2012**	Hong Kong	Cross-sectional	Survey	General public	Yes	369	Not reported	Yes	Yes	Yes	Yes
**Chang, J., 2018**	Across regions	Cross-sectional	Survey	Caregivers of children under 7 years	Yes	3358	87.4	Yes	Yes	Yes	Yes
**Gu, J., 2015**	Central	Cross-sectional	Survey	Rural and urban residents	Yes	3631	Not reported	Yes	No	Yes	Yes
**Hu, Y., 2018**	Across regions	Cross-sectional	Survey	Medical students	Yes	1819	Not reported	Yes	Yes	Yes	Yes
**Li, R., 2016**	Across regions	Cross-sectional	Survey	Guardians of children aged 0–6 years	Yes	53665	87.6	Yes	Yes	Yes	Yes
**Lv, B., 2014**	Western	Cross-sectional	Survey	Undergraduate students	Yes	731	73.1	Yes	Yes	Yes	Yes
**Pan, H., 2012**	Eastern	Cross-sectional	Survey	Shantou University (STU) students	Yes	1300	47.7 (1300/2724)	Yes	Yes	Yes	Yes
**Peng, D., 2018**	Across regions	Cross-sectional	Survey	University students in western and eastern China (Guizhou University and Zhejiang University)	Yes	3995	Not reported	Yes	Yes	No	Yes
**Wang, X., 2018**	Across regions	Cross-sectional	Survey	University students	Yes	11192	Not reported	Yes	No	Yes	Yes
**Wang, X., 2017**	Across regions	Cross-sectional	Survey	University students	Yes	11192	Not reported	Yes	Yes	Yes	Yes
**You, J. H., 2008**	Hong Kong	Cross-sectional	Survey, interview	People aged 18 or older who were uninstitutionalized Hong Kong residents	Yes	1002	14	Yes	Yes	Yes	Yes
**Yu, M., 2014**	Central	Cross-sectional	Survey	Primary caregivers	Yes	854	92	Yes	Yes	Yes	Yes
**Zhu, X., 2016**	Eastern	Cross-sectional	Survey	Jiangsu university students	Yes	660	41.6	Yes	Yes	Yes	Yes
**Jiang, H., 2017**	Eastern	Cross-sectional	Survey	Community residents in Hangzhou City	No	449	92.84	Yes	No	Yes	Yes
**Jin, Y., 2014**	Western	Cross-sectional	Survey	Middle-aged community residents	Yes	2556	98.69	Yes	No	Yes	Yes
**Li, Y., 2016**	Eastern	Cross-sectional	Survey	Community residents	Yes	1589	93.47	No	No	No	Yes
**Liao, R., 2012**	Eastern	Cross-sectional	Survey	Primary school student's parents	Yes	509	94.43	Yes	No	Yes	Yes
**Lu, T., 2016**	Eastern	Cross-sectional	Survey	University students	Yes	600	97.1 (600/618)	Yes	No	Yes	Yes
**Lv, B., 2013**	Western	Cross-sectional	Survey	University students	Yes	731	73.1	Yes	No	Yes	Yes
**Tian, L., 2015**	Eastern	Cross-sectional	Survey	University students	Yes	377	94.25	No	No	No	Yes
**Wang, J., 2017**	Central	Cross-sectional	Survey	Child parents	Yes	310	88.57	No	No	No	Yes
**Yao, Z., 2013**	Eastern	Cross-sectional	Survey	Child parents	Yes	1295	86.3	No	No	No	Yes
**Zhong, M., 2018**	Eastern	Cross-sectional	Survey	Residents	Yes	1096	90.01	No	No	No	Yes
**Dyar, O. J., 2018**	Eastern	Cross-sectional	Survey	Residents of villages	Yes	769	Not reported	Yes	Yes	Yes	Yes
**Huang, Y., 2013**	Eastern	Cross-sectional	Survey	University students	Yes	2088	83.5	Yes	No	Yes	Yes
**Lam, T. P., 2015 (BMC Pharmacol Toxicol)**	Hong Kong	Cross-sectional	Survey	Adult residents	Yes	2471	68.3	Yes	Yes	Yes	Yes
**Liao, C. C., 2006**	Taiwan	Cross-sectional	Survey	Adults over 20 years old all over Taiwan	Yes	1507	86.7 [1507/(1771-32)]	Yes	No	Yes	Yes
**Wun, Y. T., 2014**	Hong Kong	Cross-sectional	Survey	Adult residents	Yes	2471	68.3	Yes	Yes	Yes	Yes
**Chai, J., 2019**	Central	Cross-sectional	Survey, interview	Rural residents of Anhui province	Yes	2611	95	Yes	Yes	Yes	Yes
**Wang, W., 2019**	Across regions	Cross-sectional	Survey	University students	Yes	11192	Not reported	Yes	Yes	Yes	Yes
**Lam, T. P., 2003 (J Clin Pharm Ther)**	Hong Kong	Cross-sectional	Survey	Family doctors (fellows, members and	Yes	801	65.0	Yes	No	Yes	Yes

				associate members normally residing in Hong Kong)							
**Lam, T. P., 2003 (Int J Clin Pract)**	Hong Kong	Cross-sectional	Survey	Family doctors (fellows, members and associate members normally residing in Hong Kong)	Yes	801	65.0	Yes	No	Yes	Yes
**Guan, X., 2019**	Across regions	Cross-sectional	Survey	Physicians	Yes	344 questionnaires 58512 valid medical records	95.6	Yes	Yes	Yes	Yes
**Liu, C., 2019**	Central	Cross-sectional	Survey	Primary care physicians	Yes	503	71	Yes	Yes	Yes	Yes
**Lam, T. P., 2015 (Hong Kong Med J)**	Hong Kong	Cross-sectional	Survey, interview, focus group	General public	Yes	2471	68.3	Yes	No	Yes	Yes
**Wun, Y. T., 2015**	Hong Kong	Cross-sectional	Survey, interview, focus group	General public	Yes	2471	68.3	Yes	Yes	Yes	Yes
**Wun, Y. T., 2013**	Hong Kong	Cross-sectional	Survey, interview	Adult residents	Yes	2471	68.3	Yes	Yes	No	Yes
**Lu, J., 2019**	Across regions	Cross-sectional	Survey	University students	Yes	2714	Not reported	Yes	Yes	Yes	Yes
**Sun, C., 2019**	Across regions	Cross-sectional	Survey	Parents of children aged 4–13 years (who acted as the main caregiver and health decision-maker)	Yes	9526	88.7	Yes	Yes	Yes	Yes
**Xu, Y., 2020**	Across regions	Cross-sectional	Survey	Parents with children under 13 years old	Yes	6279	Not reported	Yes	Yes	Yes	Yes
**Ye, D., 2020**	Across regions	Cross-sectional	Survey	Pediatricians	Yes	472	26. 0	Yes	Yes	Yes	Yes
**Yin, J., 2019**	Across regions	Cross-sectional	Survey	Village doctors, township hospital directors	Yes	23	95.8	Yes	Yes	Yes	Yes

S5. Appraisal – qualitative (CASP)

	Section A: Are the results of the trial valid? (Yes/Can't Tell/No)						Section B: What are the results? (Yes/Can't Tell/No)			Section C: Will the results help locally? (Yes/Can't Tell/No)
**Article**	**1. Was there a clear statement of the aims of the research?**	**2. Is a qualitative methodology appropriate?**	**3. Was the research design appropriate to address the aims of the research?**	**4. Was the recruitment strategy appropriate to the aims of the research?**	**5. Was the data collected in a way that addressed the research issue?**	**6. Has the relationship between researcher and participants been adequately considered?**	**7. Have ethical issues been taken into consideration?**	**8. Was the data analysis sufficiently rigorous?**	**9. Is there a clear statement of findings?**	**10. How valuable is the research?**
**Jin, C., 2011**	Yes	Yes	Yes	Yes	Yes	No	No	Yes	Yes	Yes
**Reynolds, L., 2009**	Yes	Yes	Yes	Yes	Yes	No	Yes	No	Yes	Yes
**Zhang, Z., 2016**	Yes	Yes	Yes	Yes	Yes	Yes	Yes	Yes	Yes	Yes
**Zhu, X., 2018**	Yes	Yes	Yes	Yes	Yes	No	Yes	Yes	Yes	Yes
**Lam, T. P., 2015 (Hong Kong Med J)**	Yes	Yes	Yes	Yes	Yes	No	No	Yes	Yes	Yes
**Wun, Y. T., 2015**	Yes	Yes	Yes	Yes	Yes	No	Yes	No	Yes	Yes
**Wun, Y. T., 2013**	Yes	Yes	Yes	Yes	Yes	No	Yes	No	Yes	Yes
**Wang, X., 2020**	Yes	Yes	Yes	Yes	Yes	No	Yes	Yes	Yes	Yes
**Wei, X., 2020**	Yes	Yes	Yes	Yes	Yes	No	Yes	Yes	Yes	Yes

Lin L, *et al. BMJ Global Health* 2020; 5:e003599. doi: 10.1136/bmjgh-2020-003599

**Yin, J., 2019**	Yes	Yes	Yes	Yes	Yes	No	Yes	Yes	Yes	Yes

S6. Appraisal – mixed-methods (MMAT)

	**Screening Questions**		**1. Qualitative**					**4. Quantitative descriptive**					**5. Mixed methods**				
	S1. Are there clear research questions?	S2. Do the collected data allow to address the research questions?	1.1. Is the qualitative approach appropriate to answer the research question?	1.2. Are the qualitative data collection methods adequate to address the research question?	1.3. Are the findings adequately derived from the data?	1.4. Is the interpretation of results sufficiently substantiated by data?	1.5. Is there coherence between qualitative data sources, collection, analysis and interpretation?	4.1. Is the sampling strategy relevant to address the research question?	4.2. Is the sample representative of the target population?	4.3. Are the measurements appropriate?	4.4. Is the risk of nonresponse bias low?	4.5. Is the statistical analysis appropriate to answer the research question?	5.1. Is there an adequate rationale for using a mixed methods design to address the research question?	5.2. Are the different components of the study effectively integrated to answer the research question?	5.3. Are the outputs of the integration of qualitative and quantitative components adequately interpreted?	5.4. Are divergences and inconsistencies between quantitative and qualitative results adequately addressed?	5.5. Do the different components of the study adhere to the quality criteria of each tradition of the methods involved?
**Lam, T. P., 2015 (Hong Kong Med J)**	Yes	Yes	Yes	Yes	Yes	Yes	Yes	Yes	Yes	Yes	No	Yes	No	Yes	Yes	Yes	Yes
**Wun, Y. T., 2015**	Yes	Yes	Yes	Yes	Yes	Yes	Yes	Yes	Yes	Yes	No	Yes	No	Yes	Yes	Yes	Yes
**Wun, Y. T., 2013**	Yes	Yes	Yes	Yes	Yes	Yes	Yes	Yes	Yes	Yes	No	Yes	Yes	Yes	Yes	Yes	Yes
**Wang, X., 2020**	Yes	Yes	Yes	Yes	Yes	Yes	Yes	Yes	Yes	Yes	Yes	Yes	No	Yes	Yes	Yes	Yes
**Yin, J., 2019**	Yes	Yes	Yes	Yes	Yes	Yes	Yes	Yes	Yes	Yes	Yes	Yes	No	Yes	Yes	Yes	Yes
**Chang, J., 2017**	Yes	Yes			N/A			Yes	Yes	Yes	Yes	Yes			N/A		
**Cuurie, J., 2014**	Yes	Yes			N/A			Yes	Yes	Yes	Yes	Yes			N/A		
**Cuurie, J., 2011**	Yes	Yes			N/A			Yes	Yes	Yes	Yes	Yes			N/A		
**Xue, H., 2019**	Yes	Yes			N/A			Yes	Yes	Yes	Yes	Yes			N/A		
**Chang, J., 2019**	Yes	Yes			N/A			Yes	Yes	Yes	Yes	Yes			N/A		
**Chen, J., 2020**	Yes	Yes			N/A			Yes	Yes	Yes	Yes	Yes			N/A		

Note: Items under Q2. (quantitative randomized controlled trials) and Q3 (quantitative non-randomized) were not applicable.

S7. Included Studies

Health care users (Patients and caregivers)

1. Chai J, Coope C, Cheng J, et al. Cross-sectional study of the use of antimicrobials following common infections by rural residents in Anhui, China. BMJ Open 2019; 9(4): e024856.
2. Chan YH, Fan MM, Fok CM, et al. Antibiotics nonadherence and knowledge in a community with the world's leading prevalence of antibiotics resistance: implications for public health intervention. Am J Infect Control 2012; 40(2): 113-7.
3. Chang J, Lv B, Zhu S, et al. Non-prescription use of antibiotics among children in urban China: a cross-sectional survey of knowledge, attitudes, and practices. Expert Rev Anti Infect Ther 2018; 16(2): 163-72.
4. Dyar OJ, Yin J, Ding L, et al. Antibiotic use in people and pigs: a One Health survey of rural residents' knowledge, attitudes and practices in Shandong province, China. J Antimicrob Chemother 2018; 73(10): 2893-9.
5. Gu J, Zhao J, Huang Y, et al. Use of antibiotics by urban and rural residents in Heilongjiang Province, China: cross-sectional study. Trop Med Int Health 2015; 20(12): 1815-22.
6. Hu Y, Wang X, Tucker JD, et al. Knowledge, Attitude, and Practice with Respect to Antibiotic Use among Chinese Medical Students: A Multicentre Cross-Sectional Study. Int J Environ Res Public Health 2018; 15(6).
7. Huang Y, Gu J, Zhang M, et al. Knowledge, attitude and practice of antibiotics: a questionnaire study among 2500 Chinese students. BMC medical education 2013; 13: 163.
8. Jiang H, Jin Y, Ye J, Zhang Y, Jiang C, Zheng W. An Analysis on Knowledge, Attitude and Practice Regarding Antibiotics Use among Community Residents in Hangzhou [in Chinese]. Preventive medicine 2017; 29(10): 978-82.
9. Jin C, Ely A, Fang L, Liang X. Framing a global health risk from the bottom-up: User perceptions and practices around antibiotics in four villages in China. Health, Risk & Society 2011; 13(5): 433-49.
10. Jin Y, Lu Y, Lu P, et al. Influence of Demographic Characteristics on Antibiotics Use among Middle-Aged and Elderly People [in Chinese]. Chin J Public Health 2014; 30(09): 1140-3.
11. Lam TP, Lam KF, Ho PL, Yung RW. Knowledge, attitude, and behaviour toward antibiotics among Hong Kong people: local-born versus immigrants. Hong Kong medical journal = Xianggang yi xue za zhi 2015; 21 Suppl 7: S41-7.
12. Lam TP, Wun YT, Lam KF, Sun KS. Differences in antibiotic use between patients with and without a regular doctor in Hong Kong. BMC Pharmacol Toxicol 2015; 16: 40.
13. Li R, Xiao F, Zheng X, et al. Antibiotic misuse among children with diarrhea in China: results from a national survey. PeerJ 2016; 4: e2668.
14. Li Y, Song Y, Shao R, et al. Determinants of Self-medication with Antibiotics among Residents in Nantong [in Chinese]. The Chinese Health Service Management 2016; 33(01): 39-41.

15. Liao CC, Chang YK, Chen HH, Lu CY, Huang LY, Sung FC. Knowledge and use of antibiotics among people in Taiwan. Taiwan Journal of Public Health 2006; 25(2): 135-42.
16. Liao R. Investigation on the Impact of Parents' Cognitive Level of Antibiotics on Self-directed Use of Antibiotics in Pupils [in Chinese]. Practical Preventive Medicine 2013; 20(01): 42-5.
17. Lu J, Wang X, Lin L, Xuan Z, Hu YJ, Zhou X. The Association between Changes in External Environment Caused by Migration and Inappropriate Antibiotic Use Behaviors among Chinese University Students: A Cross-Sectional Study. Antibiotics (Basel) 2019; 8(4).
18. Lu T, Li X. A Study on the Knowledge, Attitude and Behavior of Antibiotic Use among Students in Five Universities in Nanjing [in Chinese]. ACTA UNIVERSITATIS MEDICINALIS NANJING (Social Sciences) 2016; 16(04): 274-80.
19. Lv B, Yang D, Fang Y, et al. Studying on the Behaviors and Influencing Factors of Self-Medication with Antibiotics among University Students in China [in Chinese]. 2013 Annual Meeting of Pharmacy Management Professional Committee of Chinese Pharmaceutical Association and Academic Forum on "Medicine Safety and Scientific Development"; 2013 2013/08/01; Beijing, China; 2013. p. 6.
20. Lv B, Zhou Z, Xu G, et al. Knowledge, attitudes and practices concerning self-medication with antibiotics among university students in western China. Trop Med Int Health 2014; 19(7): 769-79.
21. Pan H, Cui B, Zhang D, Farrar J, Law F, Ba-Thein W. Prior knowledge, older age, and higher allowance are risk factors for self-medication with antibiotics among university students in southern China. PLoS One 2012; 7(7): e41314.
22. Peng D, Wang X, Xu Y, Sun C, Zhou X. Antibiotic misuse among university students in developed and less developed regions of China: a cross-sectional survey. Glob Health Action 2018; 11(1): 1496973.
23. Sun C, Hu YJ, Wang X, Lu J, Lin L, Zhou X. Influence of leftover antibiotics on self-medication with antibiotics for children: a cross-sectional study from three Chinese provinces. BMJ Open 2019; 9(12): e033679.
24. Tian L, Dong J, Zeng Y, Liu W, Chang P. Logistic Regression Analysis of Factors Affecting University Students' Antibiotics Self-Medication [in Chinese]. Science Popularity (Science Education) 2015; (06): 143.
25. Wang J, Huang C, Li Z, et al. Knowledge and Behaviors of Antibiotic Use for Upper Respiratory Tract Infections among Parents of Young Children in Changsha City [in Chinese]. Chin J Public Health 2017; 33(03): 415-8.
26. Wang W, Wang X, Hu YJ, et al. The Misconception of Antibiotic Equal to an Anti-Inflammatory Drug Promoting Antibiotic Misuse among Chinese University Students. Int J Environ Res Public Health 2019; 16(3).
27. Wang X, Lin L, Xuan Z, Li L, Zhou X. Keeping Antibiotics at Home Promotes Self-Medication with Antibiotics among Chinese University Students. Int J Environ Res Public Health 2018; 15(4).

28. Wang X, Xuan Z, Storella TH, Zhou X. Determinants of non-prescription antibiotic dispensing in Chinese community pharmacies from socio-ecological and health system perspectives. Soc Sci Med 2020; 256: 113035.
29. Wun YT, Lam TP, Lam KF, Ho PL, Yung WH. The public's perspectives on antibiotic resistance and abuse among Chinese in Hong Kong. Pharmacoepidemiol Drug Saf 2013; 22(3): 241-9.
30. Wun YT, Lam TP, Lam KF, Ho PL, Yung WH. Are There Differences in Antibiotic Use Between the Recent-Immigrants from Mainland China and the Local-Born in Hong Kong? J Immigr Minor Health 2015; 17(4): 1177-84.
31. Wun YT, Lam TP, Lam KF, Sun KS. Comparison of the knowledge, attitudes and practice with antibiotic use between traditional Chinese medicine and western medicine usual attenders in Hong Kong. Complement Ther Med 2014; 22(1): 99-106.
32. Xu Y, Lu J, Sun C, Wang X, Hu YJ, Zhou X. A cross-sectional study of antibiotic misuse among Chinese children in developed and less developed provinces. J Infect Dev Ctries 2020; 14(2): 129-37.
33. Yao Z, Zhou J, Li Y, et al. Prevalence of Self-Medication with Antibiotics in Kindergarten Children of Guangzhou City [in Chinese]. Chin J Public Health 2013; 29(10): 1485-7.
34. You JH, Yau B, Choi KC, Chau CT, Huang QR, Lee SS. Public knowledge, attitudes and behavior on antibiotic use: a telephone survey in Hong Kong. Infection 2008; 36(2): 153-7.
35. Yu M, Zhao G, Stålsby Lundborg C, Zhu Y, Zhao Q, Xu B. Knowledge, attitudes, and practices of parents in rural China on the use of antibiotics in children: a cross-sectional study. BMC Infect Dis 2014; 14: 112.
36. Zhong M, Yang W, Gao G, Guan J. Analysis on Antibiotic Knowledge and Uses among the Residents of Guangdong [in Chinese]. Chin J of PHM 2018; 34(05): 589-91.
37. Zhu X, Pan H, Yang Z, Cui B, Zhang D, Ba-Thein W. Self-medication practices with antibiotics among Chinese university students. Public Health 2016; 130: 78-83.

Healthcare providers

1. Guan X, Tian Y, Song J, Zhu D, Shi L. Effect of physicians' knowledge on antibiotics rational use in China's county hospitals. Soc Sci Med 2019; 224: 149-55.
2. Lam TP, Lam KF. What are the non-biomedical reasons which make family doctors over-prescribe antibiotics for upper respiratory tract infection in a mixed private/public Asian setting? Journal of clinical pharmacy and therapeutics 2003; 28(3): 197-201.
3. Lam TP, Lam KF. Why do family doctors prescribe antibiotics for upper respiratory tract infection? International journal of clinical practice 2003; 57(3): 167-9.
4. Liu C, Liu C, Wang D, Deng Z, Tang Y, Zhang X. Determinants of antibiotic prescribing behaviors of primary care physicians in Hubei of China: a structural equation model based on the theory of planned behavior. Antimicrob Resist Infect Control 2019; 8: 23.
5. Ye D, Yan K, Zhang H, et al. A survey of knowledge, attitudes and practices concerning antibiotic prescription for upper respiratory tract infections among pediatricians in 2018 in Shaanxi Province, China. Expert Rev Anti Infect Ther 2020: 1-10.
6. Yin J, Dyar OJ, Yang P, et al. Pattern of antibiotic prescribing and factors associated with it in eight village clinics in rural Shandong Province, China: a descriptive study. Trans R Soc Trop Med Hyg 2019; 113(11): 714-21.
7. Zhu E, Fors U, Smedberg A. Understanding how to improve physicians' paradigms for prescribing antibiotics by using a conceptual design framework: a qualitative study. BMC Health Serv Res 2018; 18(1): 860.
8. Chang J, Xu S, Zhu S, et al. Assessment of non-prescription antibiotic dispensing at community pharmacies in China with simulated clients: a mixed cross-sectional and longitudinal study. The Lancet Infectious diseases 2019; 19(12): 1345-54.
9. Chang J, Ye D, Lv B, et al. Sale of antibiotics without a prescription at community pharmacies in urban China: a multicentre cross-sectional survey. J Antimicrob Chemother 2017; 72(4): 1235-42.
10. Chen J, Wang Y, Chen X, Hesketh T. Widespread illegal sales of antibiotics in Chinese pharmacies - a nationwide cross-sectional study. Antimicrob Resist Infect Control 2020; 9: 12.
11. Currie J, Lin W, Meng J. Addressing Antibiotic Abuse in China: An Experimental Audit Study. Journal of development economics 2014; 110: 39-51.
12. Currie J, Lin W, Zhang W. Patient knowledge and antibiotic abuse: Evidence from an audit study in China. Journal of health economics 2011; 30(5): 933-49.
13. Xue H, Shi Y, Huang L, et al. Diagnostic ability and inappropriate antibiotic prescriptions: a quasi-experimental study of primary care providers in rural China. J Antimicrob Chemother 2019; 74(1): 256-63.

Healthcare users and providers

1. Reynolds L, McKee M. Factors influencing antibiotic prescribing in China: an exploratory analysis. Health Policy 2009; 90(1): 32-6.

2. Wang X, Xuan Z, Storella TH, Zhou X. Determinants of non-prescription antibiotic dispensing in Chinese community pharmacies from socio-ecological and health system perspectives. Soc Sci Med 2020; 256: 113035.
3. Wei X, Deng S, Haldane V, et al. Understanding factors influencing antibiotic prescribing behaviour in rural China: a qualitative process evaluation of a cluster randomized controlled trial. J Health Serv Res Policy 2020; 25(2): 94-103.
4. Zhang Z, Zhan X, Zhou H, et al. Antibiotic prescribing of village doctors for children under 15 years with upper respiratory tract infections in rural China: A qualitative study. Medicine (Baltimore) 2016; 95(23): e3803.
